# Effects of Lipoic Acid on High-Fat Diet-Induced Alteration of Synaptic Plasticity and Brain Glucose Metabolism: A PET/CT and ^13^C-NMR Study

**DOI:** 10.1038/s41598-017-05217-z

**Published:** 2017-07-14

**Authors:** Zhigang Liu, Ishan Patil, Harsh Sancheti, Fei Yin, Enrique Cadenas

**Affiliations:** 1 College of Food Science and Engineering, Northwest A&F University, Yangling, Shaanxi 712100 China; 2 Pharmacology & Pharmaceutical Sciences, School of Pharmacy, University of Southern California, Los Angeles, CA 90089-9121 USA

## Abstract

High-fat diet (HFD)-induced obesity is accompanied by insulin resistance and compromised brain synaptic plasticity through the impairment of insulin-sensitive pathways regulating neuronal survival, learning, and memory. Lipoic acid is known to modulate the redox status of the cell and has insulin mimetic effects. This study was aimed at determining the effects of dietary administration of lipoic acid on a HFD-induced obesity model in terms of (a) insulin signaling, (b) brain glucose uptake and neuronal- and astrocytic metabolism, and (c) synaptic plasticity. 3-Month old C57BL/6J mice were divided into 4 groups exposed to their respective treatments for 9 weeks: (1) normal diet, (2) normal diet plus lipoic acid, (3) HFD, and (4) HFD plus lipoic acid. HFD resulted in higher body weight, development of insulin resistance, lower brain glucose uptake and glucose transporters, alterations in glycolytic and acetate metabolism in neurons and astrocytes, and ultimately synaptic plasticity loss evident by a decreased long-term potentiation (LTP). Lipoic acid treatment in mice on HFD prevented several HFD-induced metabolic changes and preserved synaptic plasticity. The metabolic and physiological changes in HFD-fed mice, including insulin resistance, brain glucose uptake and metabolism, and synaptic function, could be preserved by the insulin-like effect of lipoic acid.

## Introduction

Lipoic acid (1,2-dithiolane-3-pentanoic acid), a naturally occurring molecule, can be endogenously synthesized in small amounts in the mitochondria. Endogenous lipoic acid is a cofactor in energy metabolism that binds to the mitochondrial E2 subunit of α-ketoacid dehydrogenase complexes. In addition, lipoic acid modulates the redox and energy status of the cell as a function of its thiol/disulfide exchange reactions and of its ability to equilibrate between various subcellular and extracellular compartments, with ample implications for regulation of signaling and transcriptional pathways^1, 2^.

Insulin signaling plays a pivotal role in not only peripheral tissues, but also the central nervous system where it participates in neuronal survival, synaptic plasticity, memory and learning^3, 4^. The brain requires constant high supply of glucose to support its energy demanding synaptic processes to ensure cognition and memory. Neuronal energy deficits are implicated in several neurodegenerative disorders^5, 6^. The redox-energy modulating properties of lipoic acid are thought, in part, to be involved in its ability to reduce insulin resistance in animal models and humans^7–9^. Previous studies demonstrated that lipoic acid could restore the impairment of brain glucose uptake, mitochondrial bioenergetics, and synaptic plasticity in the aging brain through the modulation of insulin signaling^10^. Studies on a mouse model of Alzheimer’s disease (3 × TG-AD) revealed that lipoic acid administration modulated synaptic plasticity through an insulin-like effect, probably involving redox modulation upstream of PI3K, *i.e*., at the IRS level. ^13^C-NMR studies showed that the metabolic deficits inherent in 12 month-old 3 × TG-AD mice were reversed by lipoic acid, whereas lipoic acid prevented the hypermetabolism observed in the 3 × TG-AD mice at 6-month-of-age^11–13^.

High dietary fat intake is associated with several metabolic diseases under the umbrella of metabolic syndrome, such as obesity and type 2 diabetes. High-fat diet (HFD) feeding in rodent models induced brain insulin resistance and further impaired synaptic plasticity^14, 15^. Studies in different experimental models have demonstrated that lipoic acid had positive effects on decreasing body weight^16^, reversing insulin resistance^17^, and attenuating HFD-induced oxidative damages in liver^18, 19^ and in the central nervous system, lipoic acid exhibited beneficial effects against HFD-induced spatial learning impairments^20^. More recently, it was shown that lipoic acid improved neuronal insulin signaling and rescued cognitive function in high fat-fed rats, effects ascribed to the lipoic acid-mediated increase in vesicular glutamate transporters^21^.

The present study assesses the effects of lipoic acid on HFD-induced alteration of brain insulin signaling, glucose uptake, and synaptic plasticity by examining glucose transporters activation combined with [^18^F]-fluorodeoxyglucose (FDG) positron emission tomography/Computerized tomography (PET/CT) scanning, the insulin-sensitive PI3K/Akt pathway, *ex vivo* ^13^C NMR assessment of neuronal and astrocytic glycolytic activity and TCA cycle^22^, and –as a functional outcome– long-term potentiation (LTP).

## Results

### Metabolic changes in HFD-fed mice and the effect of lipoic acid

**Figure 1:**
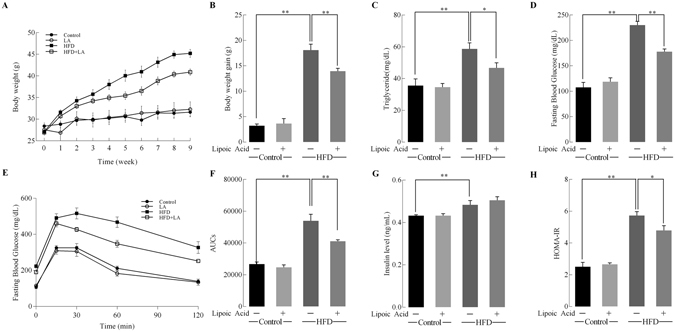
Effects of lipoic acid and HFD on body weight gain and insulin resistance. Mice were fed with 9-week HFD or normal diet, with or without lipoic acid administration in drinking water (0.23% w/v) and different parameters were monitored weekly or at the end of the 9-week period. (**A**) Body weight changes; (**B**) Body weight gain; At the end of the study (week 9) the following parameters were examined: (**C**) Plasma triglyceride levels; (**D**) Fasting glucose level; (**E**) Glucose tolerance test; (**F**) Area-under the curve values of glucose tolerance test; (**G**) Fasting insulin levels; (**H**) Insulin resistance index, HOMA-IR. Data presented as mean ± SD, *n* ≥ 5, *p < 0.05, **p < 0.01.

3-Month old C57BL/6J mice were randomized into four groups (*n* = 10 per group): (1) control diet, (2) control diet plus lipoic acid (0.23% w/v lipoic acid in drinking water), (3) HFD, and (4) HFD plus lipoic acid for 9 weeks. All the diet factor, lipoic acid treatment, and their interaction has significant effects on the bodyweight gain [F(1, 36) = 9.608 p = 0.0042, F(1, 36) = 6.213 p = 0.0184, F(1, 36) = 319.0 p < 0.0001, respectively]. Consistent with previous studies, the HFD feeding induced an increase in body weight from week 2 on (17.5% higher compared to control group); this pattern was observed for the rest of the HFD feeding period (40.1% higher at week 9) (Fig. 1A). The HFD + lipoic acid group showed a significantly (p < 0.01) lower body weight gain (22.9%) when compared with HFD group (Fig. 1B), but no significant differences on energy intake (supplemental Fig. 1).

Hyperlipidemia and hyperglycemia developed after HFD feeding for 9 weeks as shown by fasting triglyceride levels and glucose levels (64.8% and 113.4% higher than the control group, respectively) (Fig. 1C and D). However, the HFD + lipoic acid group showed 20.4% and 22.6% lower fasting blood triglyceride and glucose levels, respectively, compared to the HFD group (Fig. 1C and D). All the diet factor, lipoic acid treatment, and their interaction has significant effects on the glucose tolerance [F(1, 16) = 6.752 p = 0.0202, F(1, 16) = 12.73 p = 0.0028, F(1, 16) = 109.4 p < 0.0001, respectively]. The area-under the curve values indicated that lipoic acid treatment significantly ameliorated HFD-induced glucose intolerance (Fig. 1E and F). The HFD-fed mice also showed development of hyperinsulinemia at the end of week 9 with 11.6% higher fasting insulin levels whereas the lipoic acid-supplemented HFD group did not show any significant differences in fasting insulin levels (Fig. 1G). The HFD-induced hyperglycemia and hyperinsulinemia was reflected by the HOMA-IR (homeostatic model assessment - insulin resistance) index at the end of week 9 (more than 2-fold over the control group value), which is consistent with our previous study^14^. Similar to the fasting plasma glucose, the HOMA-IR was markedly lower than that of HFD + lipoic acid group compared to the HFD group (Fig. 1H).

### Effects of lipoic acid on brain glucose uptake

**Figure 2:**
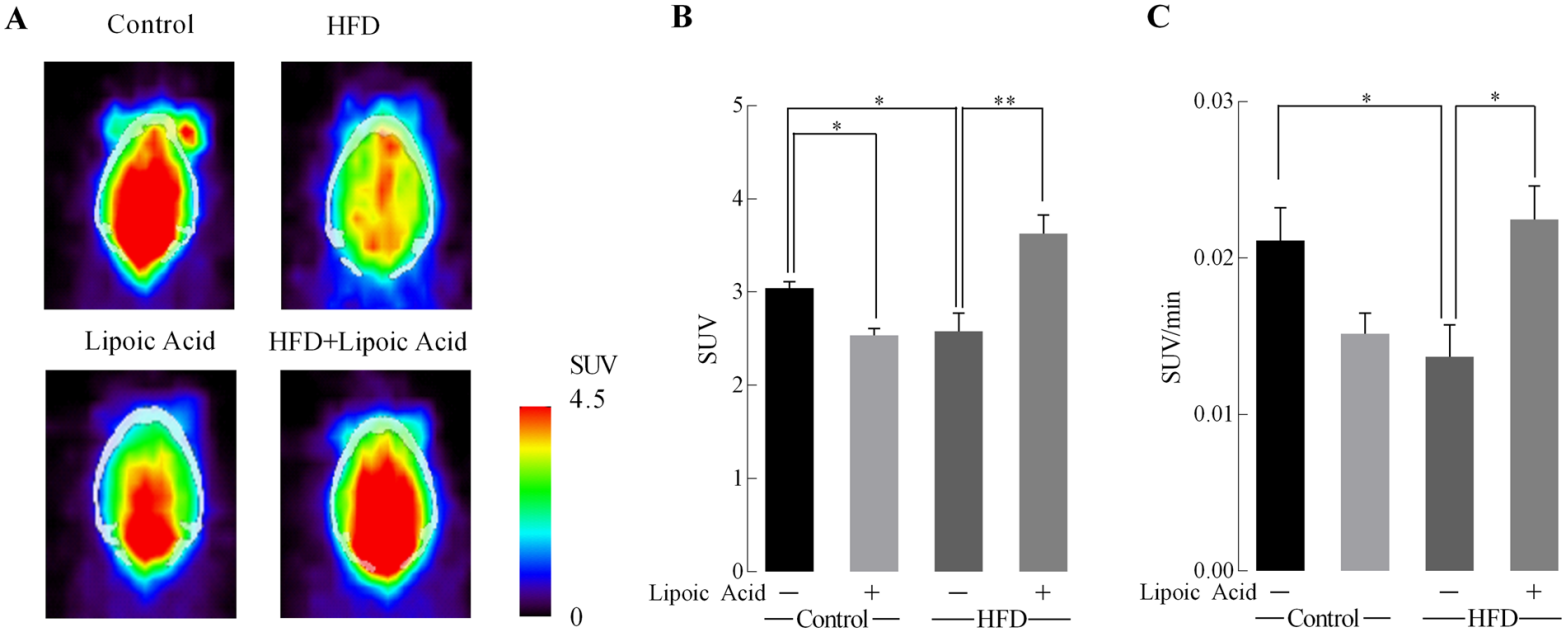
Effect of lipoic acid on brain glucose uptake. (**A**) Representative images of brain glucose uptake ([^18^F]-FDG microPET/CT) in control, lipoic acid, HFD, HFD + lipoic acid groups at the last scanning point; (**B**) Glucose standard uptake values (SUV) and (**C**) glucose standard uptake rate (SUV/time). Data presented as mean ± SD, *n* ≥ 5, *p < 0.05, **p < 0.01.

Figure 2A shows [^18^F]-FDG-PET (dynamic microPET scanning) images of the four aforementioned groups at the end of week 9. Standardized glucose uptake values (SUV) were measured at different time points. The diet × lipoic acid treatment interaction had significant effects on SUV values [F(1, 16) = 34.7, p < 0.0001]. The SUV were significantly (p < 0.05) lower in the brains of the HFD group than it’s in the control diet group. Lipoic acid administration prevented these decreases in SUV and SUV/min in the HFD group. Control mice fed lipoic acid also showed an unexpected statistically significant lower value of SUV but there was no statistically significant difference in value of SUV/min between these two groups (p < 0.05) (Fig. 2B and C **)**.

### Effects of lipoic acid on brain glucose transporter function and the PI3K/Akt signaling pathway

**Figure 3:**
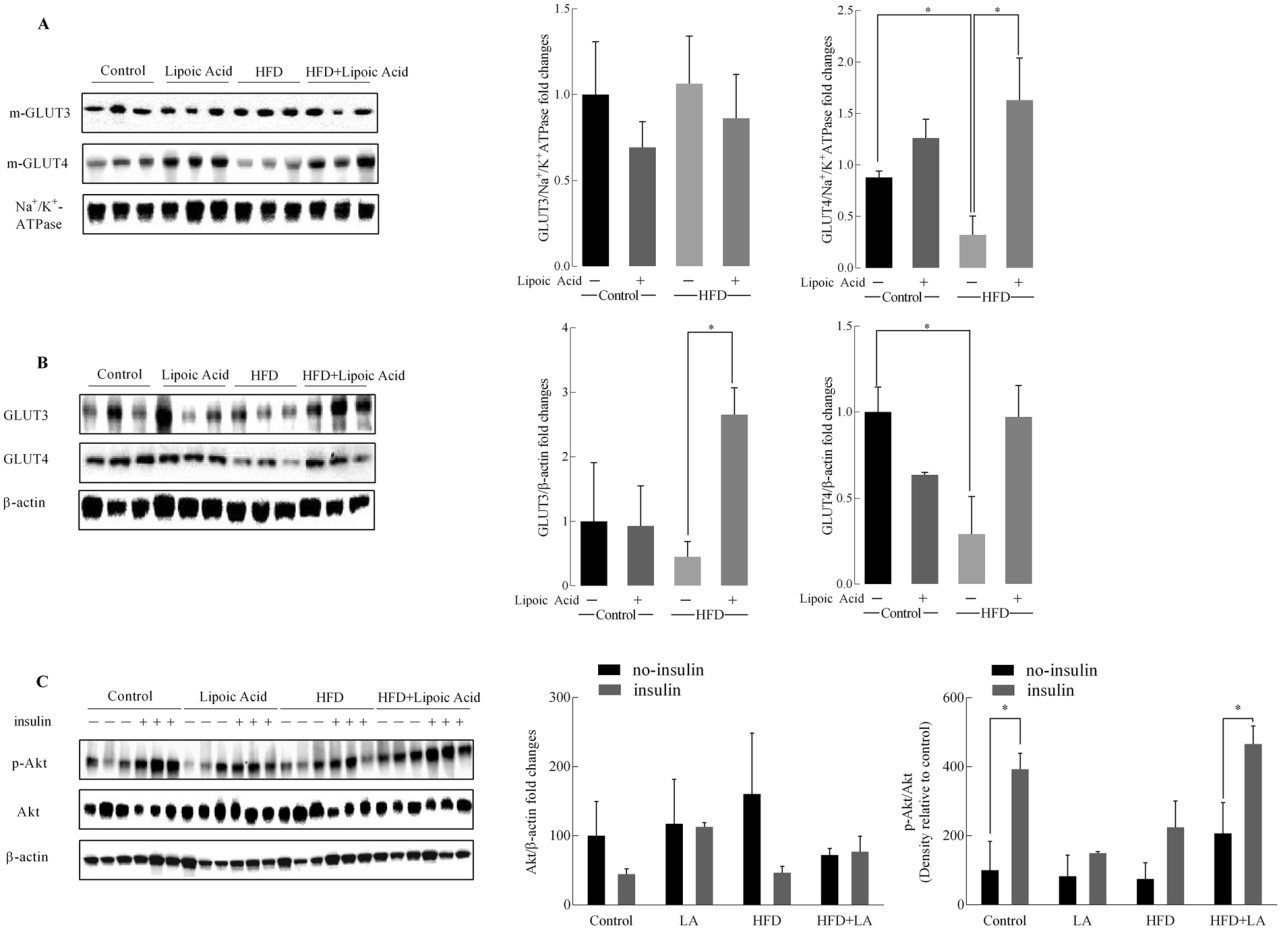
Effect of lipoic acid on HFD-induced alterations in the expression of glucose transporters and the insulin signaling. Expression of (**A**) membrane associated GLUT3 and GLUT4; (**B**) Total levels of GLUT3 and GLUT4. (**C**) Representative western blots of brain tissue from control, +lipoic acid, HFD, and HFD + lipoic acid with (+) or without (−) *ex vivo* insulin stimulation. Na^+^/K^+^ ATPase and β-actin were used as loading control for membrane fraction and whole homogenate. Data presented as mean ± SD respectively, *n* ≥ 3 animals, *p < 0.05, **p < 0.01.

Brain glucose transporters facilitate the supply of glucose into neural cells like astrocytes and neurons after crossing the blood-brain-barrier (BBB). Total- and cell membrane-localized neuronal glucose transporters, GLUT3 and GLUT4 are shown in Fig. 3A and B. GLUT3 and GLUT4 in the membrane fraction are the active and functional forms of these transporters. There were no statistically significant changes either in the membrane-bound GLUT3 or total protein GLUT3 in the control and HFD groups; lipoic acid induced a significant (*P* < 0.01) increase in the total expression of GLUT3 in HFD mouse brains (Fig. 3B) but not its translocation to the membrane (Fig. 3A). Translocation of GLUT4 to the plasma membrane is promoted by activation of insulin signaling^23^. The total- and membrane levels of the insulin-sensitive and neuron-specific GLUT4 decreased sharply in the HFD group; these effects were prevented by lipoic acid treatment (Fig. 3A and B). ANOVA analyses indicated that the lipoic treatment significantly decreased total GLUT4 protein levels [F(1, 8) = 6.936, p = 0.0389]. The diet × lipoic acid treatment interaction prevented the decrease in total- and cell membrane-localized GLUT4 protein levels induced by HFD [F(1, 6) = 28.82, p = 0.0017, F(1, 8) = 7.933, p = 0.0226, respectively].

Insulin affects neuronal survival and plasticity through the PI3K/Akt branch of the insulin signaling^4^. Brain insulin sensitivity (Fig. 3C) was further determined in brain slices with and without insulin stimulation: control mice showed a remarkable increase in both p-Akt/Akt and Akt/β-actin ratios upon insulin stimulation, while there were no significant differences of Akt/β-actin ratios between the insulin treatment and non-insulin treatment brain tissues. (Fig. 3C). Diet and lipoic acid supplementation has no significant influence on Akt expression. Consistent with the insulin resistance inherent in the HFD group (Fig. 1F **)**, insulin did not induce a significant activation of Akt in this group. However, lipoic acid administration led to a substantial increase in Akt activation (p-Akt/Akt) upon insulin stimulation in the HFD group, but it did not change expression of AKT (Fig. 3C).

### Neuronal versus glial metabolism: NMR analysis of ^13^C metabolite concentration with [1–^13^C]-Glucose and [1,2-^13^C]-acetate infusion

The co-infusion of ^13^C-labeled glucose and acetate lead to a typical labeling pattern that can be directly quantified by NMR spectroscopy^13, 24^. While glucose is utilized by both neurons and astrocytes, acetate is exclusively metabolized by the latter^22^. The simultaneous intravenous infusion of [1-^13^C]-Glucose and [1,2-^13^C]-acetate and the following NMR quantification of their different isotopomers and ^13^C flux allows for the assessment of metabolic changes in neurons versus those in astrocytes.

The concentration of ^13^C-labeled Glu (glutamate), Gln (glutamine), Asp (aspartate), γ-aminobutyric acid (GABA), N-acetylaspartate (NAA), lactate (Lac), and alanine (Ala) isotopomers in brains from all experimental groups are shown in Table 1. The concentrations of [3-^13^C]-Ala, [4,5-^13^C]-Gln, [2,3 and 3,4-^13^C]-Gln, [3-^13^C]-GABA, [2-^13^C]-GABA, and [4-^13^C]-Asp were significantly higher in HFD-feeding mice brain than those in control group, while lipoic acid supplementation in the HFD group preserved the levels of [3-^13^C]-Ala, [3-^13^C]-GABA, [2-^13^C]-GABA, and [4-^13^C]-Asp.

**Table 1:** Concentration of the different isotopomers of ^13^C labeled Ala, Lac, Glu, Gln, Asp, GABA, MI, and NAA after [1-^13^C]-Glucose +[1, 2-^13^C]-Acetate infusion.

Metabolites	Control	LA	HFD	HFD + LA
[3-^13^C]-Ala	0.07 ± 0.01	0.08 ± 0.02	0.13 ± 0.00^*^	0.08 ± 0.01^#^
[3-^13^C]-Lac	1.42 ± 0.07	1.22 ± 0.19	1.48 ± 0.03	1.23 ± 0.10
[4-^13^C]-Glu	0.72 ± 0.04	0.65 ± 0.06	0.68 ± 0.04	0.57 ± 0.04
[3-^13^C]-Glu	0.44 ± 0.04	0.38 ± 0.05	0.47 ± 0.02	0.42 ± 0.03
[2-^13^C]-Glu	0.52 ± 0.03	0.46 ± 0.05	0.54 ± 0.01	0.50 ± 0.02
[4,5-^13^C]-Glu	0.22 ± 0.02	0.17 ± 0.01	0.24 ± 0.02	0.21 ± 0.01
[1,2-^13^C]-Glu	0.11 ± 0.01	0.08 ± 0.01	0.12 ± 0.01	0.12 ± 0.01
[2,3-^13^C]-Glu	0.14 ± 0.01	0.13 ± 0.01	0.14 ± 0.01	0.13 ± 0.02
[2,3-^13^C]-Glu^#^	0.25 ± 0.01	0.22 ± 0.00	0.25 ± 0.02	0.20 ± 0.01
[4-^13^C]-Gln	0.22 ± 0.02	0.20 ± 0.04	0.27 ± 0.02	0.24 ± 0.01
[3-^13^C]-Gln	0.18 ± 0.02	0.17 ± 0.02	0.23 ± 0.01	0.19 ± 0.01
[2-^13^C]-Gln	0.16 ± 0.03	0.16 ± 0.03	0.19 ± 0.01	0.16 ± 0.02
[4,5-^13^C]-Gln	0.27 ± 0.04	0.19 ± 0.04	0.40 ± 0.03^**^	0.37 ± 0.02
[1,2-^13^C]-Gln	0.07 ± 0.01	0.07 ± 0.01	0.10 ± 0.01	0.07 ± 0.01
[2,3-^13^C]-Gln	0.05 ± 0.01	0.04 ± 0.01	0.06 ± 0.00	0.07 ± 0.01
[2,3-^13^C]-Gln^#^	0.09 ± 0.01	0.09 ± 0.01	0.15 ± 0.01^**^	0.12 ± 0.01
[4-^13^C]-Asp	0.11 ± 0.01	0.06 ± 0.03	0.18 ± 0.02^*^	0.08 ± 0.01^#^
[3-^13^C]-Asp	0.19 ± 0.01	0.17 ± 0.02	0.18 ± 0.00	0.18 ± 0.01
[2-^13^C]-Asp	0.21 ± 0.00	0.19 ± 0.02	0.20 ± 0.00	0.19 ± 0.01
[1-^13^C]-Asp	0.08 ± 0.02	0.10 ± 0.02	0.13 ± 0.01	0.11 ± 0.03
[2,3-^13^C]-Asp	0.05 ± 0.01	0.03 ± 0.01	0.05 ± 0.00	0.04 ± 0.01
[3,4-^13^C]-Asp	0.06 ± 0.03	0.04 ± 0.01	0.04 ± 0.00	0.03 ± 0.01
[4-^13^C]-GABA	0.12 ± 0.01	0.10 ± 0.02	0.12 ± 0.01	0.11 ± 0.01
[3-^13^C]-GABA	0.32 ± 0.04	0.32 ± 0.03	0.59 ± 0.05^**^	0.31 ± 0.04^##^
[2-^13^C]-GABA	0.32 ± 0.05	0.39 ± 0.09	0.61 ± 0.06^*^	0.33 ± 0.04^#^
[1-^13^C]-GABA	0.07 ± 0.01	0.07 ± 0.02	0.09 ± 0.01	0.05 ± 0.01
[1,2-^13^C]-GABA	0.07 ± 0.01	0.04 ± 0.01	0.07 ± 0.00	0.06 ± 0.01
[2,3-^13^C]-GABA	0.05 ± 0.02	0.03 ± 0.01	0.05 ± 0.01	0.04 ± 0.01
[3,4-^13^C]-GABA	0.02 ± 0.01	0.01 ± 0.00	0.03 ± 0.00	0.02 ± 0.01
[4,6-^13^C]-MI	0.08 ± 0.01	0.09 ± 0.01	0.10 ± 0.01	0.09 ± 0.01
[2-^13^C]-MI	0.04 ± 0.01	0.03 ± 0.01	0.03 ± 0.00	0.05 ± 0.01
[1,3-^13^C]-MI	0.08 ± 0.02	0.07 ± 0.01	0.08 ± 0.01	0.08 ± 0.01
[5-^13^C]-MI	0.06 ± 0.01	0.04 ± 0.01	0.05 ± 0.01	0.07 ± 0.01
[6-^13^C]-NAA	0.05 ± 0.01	0.04 ± 0.01	0.05 ± 0.01	0.05 ± 0.01
[3-^13^C]-NAA	0.08 ± 0.01	0.05 ± 0.01	0.07 ± 0.00	0.06 ± 0.00
[2-^13^C]-NAA	0.04 ± 0.01	0.03 ± 0.00	0.03 ± 0.00	0.02 ± 0.01

Concentration of the isotopomers of ^13^C g Glu, Gln, Asp, GABA, MI, and NAA in mice fed standard/HFD with/without lipoic acid, after 60 min of [1-^13^C]-Glucose+[1, 2-^13^C]-Acetate infusion. Results in the columns 2–5 are presented as average nmol/mg brain tissue ± SEM; *p ≤ 0.05 **p ≤ 0.01 versus control group, ^#^ *p* ≤ 0.05 ^##^ *p* ≤ 0.01 versus HFD group; *n* ≥ 4 per group. The results for Glu, Gln, GABA, and Asp with single ^13^C are corrected for natural abundance (the results for Ala, Lac, MI, NAA, and with ^13^C doublets are not corrected for natural abundance). [2,3-^13^C]-Gln/Glu^#^ stand for [2,3 and 3,4-^13^C]-Gln/Glu, respectively.

**Figure 4:**
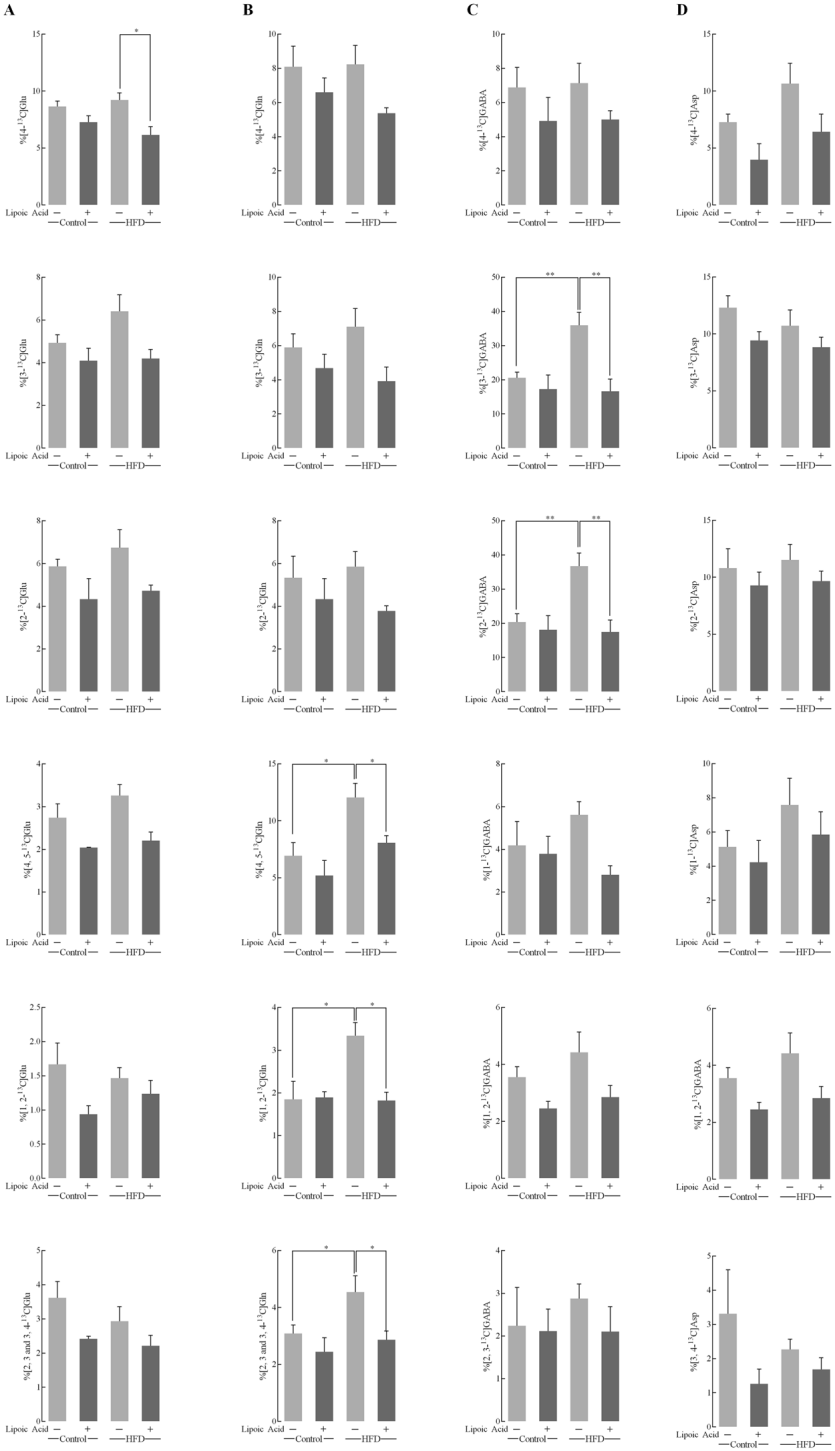
^13^C enrichment percentage of the major metabolites isotopomers after [1-^13^C]-glucose and [1,2-^13^C]-acetate infusion. These 18- bar graph show the different ^13^C labeled glutamate (column **A**), glutamine (column B), GABA (column **C**), and Aspartate (column **D**) isotopomers after infusion of [1-^13^C]-glucose+[1,2-^13^C]-acetate for 60 min. The enrichment of each metabolite isotopomers was calculated as described in Materials and methods section. Data presented as mean ± SD, *n* ≥ 4, *p < 0.05, **p < 0.01.

**Figure 5:**
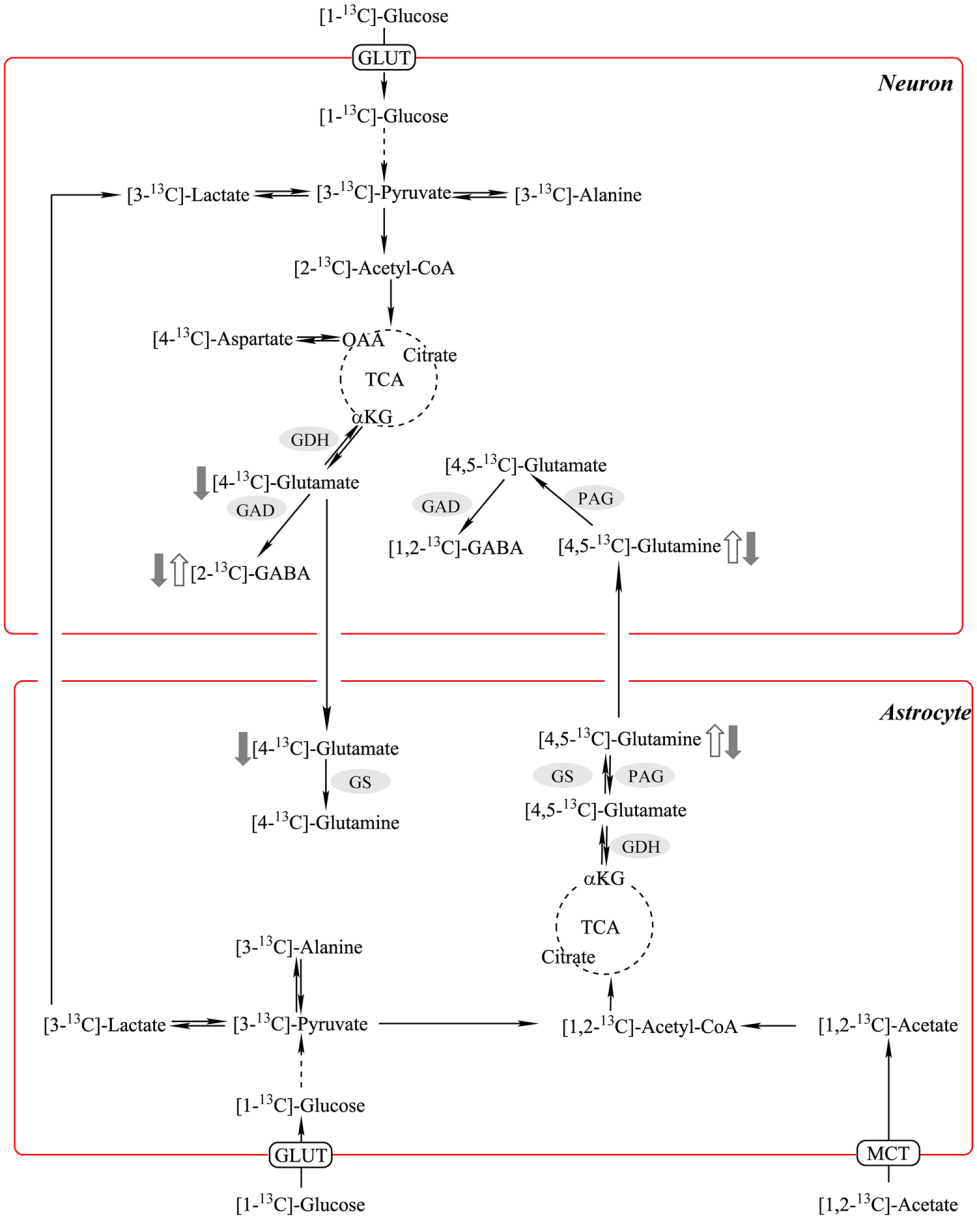
Neuronal–astrocytic metabolic interactions. Labeling pattern after [1-^13^C]-glucose+[1,2-^13^C]-acetate infusion. Effect of HFD on enrichments of different isotopomers indicated by open arrows; effect of lipoic acid on enrichments of different isotopomers on HFD-feeding mice indicated by close arrows.

The enrichments of these metabolites (calculated as Materials and methods section) reflect the relative content of different isotopomers enriched in TCA cycles. Figure 5 illustrated the typical labeling pattern after [1-^13^C]-Glucose+[1,2-^13^C]-Acetate infusion metabolites in the first turn of TCA cycle. The total concentrations [^12^C + ^13^C] of glutamate, glutamine, GABA, and aspartate were analyzed by HPLC, suggested little changes in all four metabolites across all groups (Supplemental Fig. 2). The fractional enrichment of various metabolite isotopomers of glutamate, glutamine, GABA, and aspartate (Fig. 4) were quantified based on the absolute concentrations shown in Table 1. The fractional enrichments reflect the metabolism state of mice brain. The HFD group showed statistically significant higher levels of [4,5-^13^C]-Gln, [1,2-^13^C]-Gln, [2,3 and 3,4-^13^C]-Gln, [3-^13^C]-GABA, and [2-^13^C]-GABA (Fig. 4). Lipoic acid supplementation in the HFD group showed lower levels of [4-^13^C]-Glu, [4,5-^13^C]-Gln, [1,2-^13^C]-Gln, [2,3 and 3,4-^13^C]-Gln, [3-^13^C]-GABA, and [2-^13^C]-GABA (Fig. 4).

### Metabolic ratios in HFD-feeding mice with/without lipoic acid

**Figure 6:**
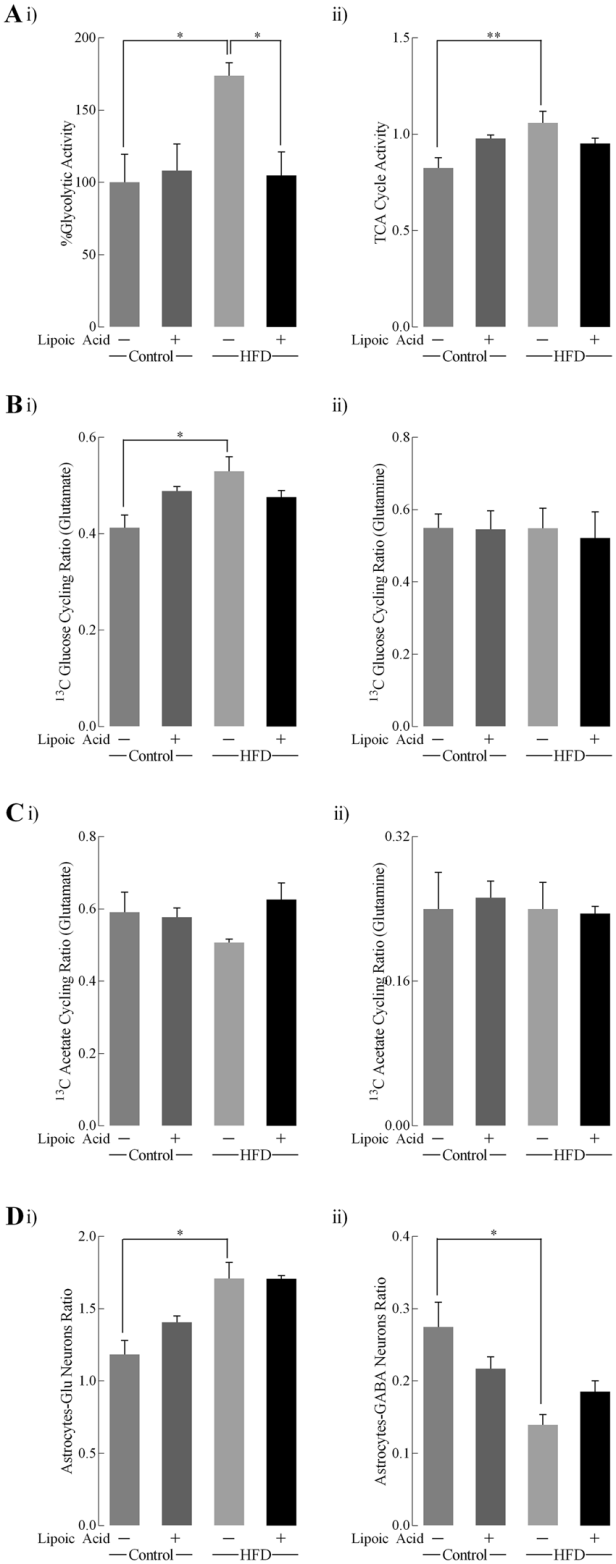
Metabolic ratios quantified after [1-^13^C]-glucose+[1,2-^13^C]-acetate infusion. Metabolic ratios were calculated: (**Ai**) Percentage of glycolytic activity was calculated from the levels of [3-^13^C]-alanine; (**Aii**) TCA cycle activity was based on glutamate formation from [1-^13^C]-glucose; (**Bi** and **Bii**) ^13^C glucose cycling ratio for glutamate and glutamine respectively; (**Ci** and **Cii**) ^13^C acetate cycling ratio for glutamate and glutamine, respectively; (**Di**) transfer of glutamine from astrocytes to glutamatergic neurons, and (**Dii**) transfer of glutamine to GABAergic neurons. Data presented as mean ± SD, *n* ≥ 4, *p < 0.05, **p < 0.01.

Metabolic ratios were calculated based on the concentration of different isotopomers after infusion ^13^C-labeled glucose and acetate^13, 24^. The calculation of these metabolic ratios was followed the formulas provided in *Materials and Methods* section. As shown in Fig. 6A and B, the percentage of glycolytic activity (measured from alanine levels) and TCA cycle activity in the HFD group was significantly higher than that in the control diet group, which indicated a hypermetabolic state in brain. Lipoic acid significantly (p < 0.05) lowered the glycolytic activity of HFD group to a level comparable to the control group, while it had little effect on lowering the TCA cycle activity. The transfer ratio represents the substrate (glutamine) transfer from astrocytes to the specific neuron types^24^. Glutamine transfer to glutamatergic neurons was significantly increased by HFD feeding, while the transfer of glutamine to GABAergic neurons was lower in HFD group (Fig. 6D). Lipoic acid did not alter the glutamine transfer into the glutamatergic or GABAergic neurons.

### Lipoic acid and hippocampal synaptic plasticity

**Figure 7:**
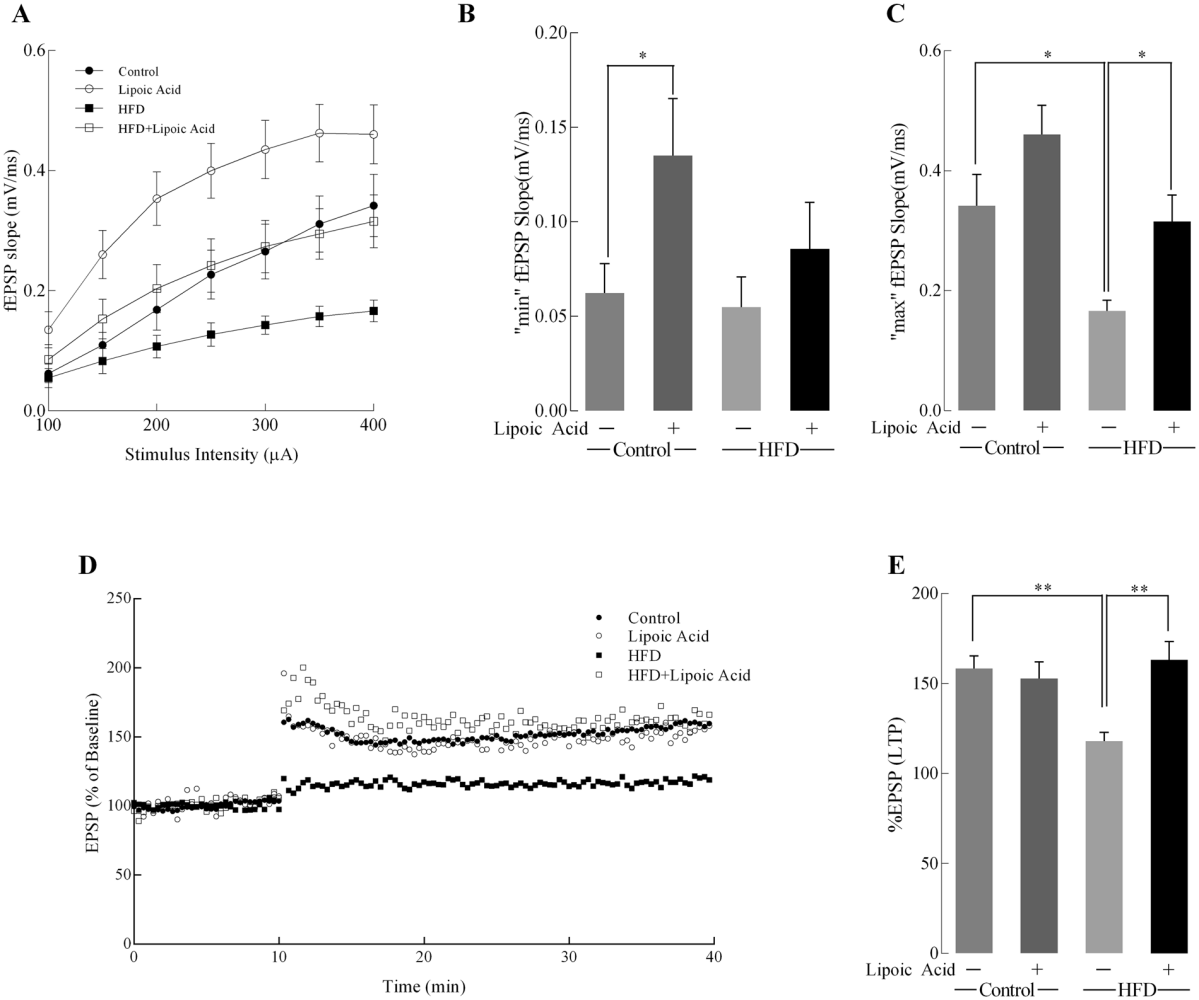
Effects of lipoic acid on hippocampal synaptic plasticity. Input/Output (I/O) and changes after induction of LTP in all groups. (**A**) I/O relationship curves at increasing stimulus intensities; Bar graphs showing the minimum (**B**) and maximum (**C**) fEPSP slope values at 100 and 400 µA; (**D**) Baseline fEPSP slopes and those after induction of LTP; (**E**) The measured LTP using %EPSP for the last 5 min of the response to TBS stimulation. For each sub-figure: total *n* ≥ 8 slices/group and at least 3–4 animals/group. Data presented as mean ± SD, *n* ≥ 5, *p < 0.05, **p < 0.01.

Insulin signaling plays a critical role in regulating brain synaptic plasticity via modulation of LTP) and long-term depression (LTD)^4^. Electrophysiology was used to assess synaptic plasticity in the hippocampal CA1 region by examining the Input/Output (I/O) responses, which indicate the strength of synaptic transmission and the connections between neurons. The HFD feeding led to a substantial lower value of I/O response relative to the control group, whereas the HFD group treated with lipoic acid resulted in an I/O response similar to that observed in the control group (Fig. 7A). Diet factor and lipoic treatment factor had significant effects on maximum output values [F(1, 43) = 6.945 p = 0.069, F(1, 43) p = 0.005, respectively]. The decrease of minimum output (Fig. 7B) in the HFD group was not statistically significant; however, the maximum output (Fig. 7C) was significantly decreased (51.2%, p < 0.05) compared with mice on the control diet. Lipoic acid treatment alone improved the minimum output but not maximum value in control diet-fed mice, while it elevated the levels of the maximum output in the HFD-fed mice brain. All the diet factor, lipoic acid treatment, and their interaction has significant effects on LTP values [F(1, 243) = 118.3 p < 0.0001, F(1, 16) = 71.34 p < 0.0001, F(1, 16) = 41.40 p < 0.0001, respectively]. The HFD group manifested a substantially reduced LTP as compared to control group by 25.5%, which was prevented by lipoic acid treatment (Fig. 7D and E). These data showed that lipoic acid has protective effects on HFD-elicited deficits in hippocampal LTP.

## Discussion

This study assessed the effects of lipoic acid on brain insulin signaling, glucose metabolism, and synaptic plasticity in a mouse model of HFD-induced obesity. Previous research showed that high-fat diet impaired brain energy metabolism, and consequently triggered decreased synaptic plasticity and cognitive function defects^14, 25^. Consistently, the HFD fed mice in this study showed development of insulin resistance, decreased brain glucose uptake, development of a brain hypermetabolic state, and decreased synaptic plasticity. Treatment with lipoic acid preserved insulin sensitivity, insulin-signaling activity, and maintained synaptic plasticity and prevented the development of the hypermetabolic state in the HFD group.

Clinical trials assessing the effects of lipoic acid on obesity, diabetes, diabetic neuropathies, cardiovascular disease, and Alzheimer’s disease patients indicated that dietary supplementation of lipoic acid had beneficial effects on plasma lipid disorders, inflammatory responses, oxidative stress, and stabilized or slowed the cognitive decline in Alzheimer’s patients^26–32^. These clinical trials support the translatability of this research and neuroprotective effects of lipoic acid afforded by oral intake. In this work, lipoic acid was administered in the drinking water at a concentration of 0.23% w/v; orally administered lipoic acid was observed in the rat brain^33, 34^ although its level in brain was quantified as extremely low^35^.

Insulin signaling controls cellular metabolism of central nervous system via mediating glucose uptake and mitochondrial metabolic function^4^. Lipoic acid ameliorated HFD-induced brain insulin resistance via stimulation of the PI3K/Akt pathway. Lipoic acid treatment alone had no effect on Akt phosphorylation in the group fed a control diet. A similar scenario has also been observed in our previous study in which lipoic acid treatment only elevated Akt phosphorylation in the brains of 24-month-old rats but not in that of 6- or 12-month-old rats^36^. Lipoic acid treatment was able to preserve insulin-sensitivity (Fig. 3) and the rate of glucose uptake (Fig. 2) as well as maintain brain glucose metabolism in HFD-fed mice. Lipoic acid exerts its insulin-mimetic actions through thiol/disulfide exchange reactions, which allows it to regulate the function of key cysteine-rich proteins of the insulin pathway, primarily the insulin receptor and insulin receptor substrate (IRS)^37^. Interestingly, it was observed that lipoid acid in control-diet mice elicited a decreased brain glucose update value (SUV, Fig. 2B), which requires further investigation for underlying mechanisms. However, the lack of effect of lipoic acid on control diet group in terms of the glucose standard uptake rate (SUV/min, Fig. 2C), the expression of total and membrane-associated GLUTs, the insulin signaling and the LTP suggested that lipoic acid has minimum functionally negative effect on brain metabolism and synaptic function in the control diet group. Nevertheless, lipoic acid treatment prevented HFD-induced glucose uptake defects indicating its pivotal role in regulating glucose metabolism in the brain.

A co-infusion of [1-^13^C]-glucose and [1,2-^13^C]-acetate and the NMR-spectroscopy quantification was used to assess the contribution of neurons and astrocytes to metabolic profile in the brain. In the HFD-mice brain, an overall hypermetabolic state was evidenced by (a) increased glycolytic activity indicated by alanine concentration, (b) increased activity of TCA cycle (Fig. 6), and (c) an overall increase in enrichment of ^13^C labeled metabolites (Fig. 4). However, the mice exhibited reduced brain glucose uptake (Fig. 2) and decreased insulin signaling (Fig. 3), indicative of brain insulin resistance. The discrepancy between decreased substrate supply (*i.e*., reduced brain glucose uptake) and increased energy metabolism can be bridged by assessing the differences in neuronal and astrocytic glucose uptake: resistance to insulin in the brain will primarily affect the insulin-sensitive GLUT4 (Fig. 3A and C), which is primarily expressed in selective neurons in the hippocampus, cortex, hypothalamus, and olfactory bulbs^38^. The total expression and translocation of GLUT4 is known to be reduced upon hyperglycemia^14^. Therefore, it can be hypothesized that the majority of glucose in the bloodstream is taken up by astrocytic GLUTs (Fig. 5 **)**, where it is metabolized to generate lactate and glutamine, which are released into the extracellular space in order to provide the neurons with energy substrates. Additionally, astrocytic metabolism is supported by astrocyte-specific ketogenic substrate like acetate (used in ^13^C co-infusion with glucose). However, lipoic acid had no effects on the translocation of another insulin-sensitive glucose transporter GLUT3^39^. GLUT8, also known as SLC2A8, is a recently-discovered neuronal glucose transporter, whose translocation to the membrane is also insulin sensitive^40^. Whether or not lipoic acid plays a role in regulating GLUT8 in central nervous system needs to be further investigated.

Astrocytes are known to support neuronal metabolic requirements, with astrocytic glutamine used by neurons to synthesize glutamate and GABA^41^. Interestingly, the transfer of [4,5-^13^C]-glutamine (synthesized in astrocytes from [1,2-^13^C]-acetate) to neurons was substantially increased in the HFD-fed group (Fig. 6Di). [3-^13^C]-lactate, produced in astrocytes from [1-^13^C]-glucose, can be transferred to neurons and converted to [3-^13^C]-pyruvate, the further metabolism of which gives rise to the increased [2-^13^C]-GABA levels observed in samples from the HFD group. Compared to neurons, astrocytes also present a higher glycolytic rate and lower oxidative phosphorylation rate^42^. When all these factors are taken into account, it can be surmised that the hypermetabolic state observed in the brains of the HFD-mice, is a result of increased astrocytic metabolic activity, in order to satisfy the bioenergetics demands of the insulin-resistant neurons. In the lipoic acid-supplemented HFD group, the levels of total and ^13^C-glucose metabolites and rate of metabolism were brought down to those observed in the control mice. Lipoic acid was able to reduce significantly the enrichment of [4,5-^13^C]-glutamine, [4-^13^C]-glutamate, and [4-^13^C]-glutamine, and decreased levels of [2-^13^C]-GABA. Additionally, lipoic acid feeding decreased glycolytic activity and increased the ^13^C-acetate cycling ratio, as compared to the HFD-mice that were not fed lipoic acid. These observations indicated that lipoic acid was able to preserve neuronal glucose metabolism, and possibly even prevented metabolic hyper-activity of astrocytes. The functional effects of lipoic acid could be an adaptive response to the redox environment by virtue of its thiol-disulfide exchange properties^37^.

The memory of an event is stored by the brain through the process of modifying neuronal networks, through LTP and LTD (Long-term depression). LTP occurs when a pre-synaptic neuron repetitively and prolongedly excites a post-synaptic neuron, leading to the depolarization of the latter being maintained for a longer period of time, ultimately leading to formation of new synapses^43^. LTP in the hippocampal CA1 region is a key form of plasticity accounting for learning and memory. HFD brains elicited decreased LTP relative to the control group. This impairment in synaptic plasticity could be a result of (*a*) the inability of the now insulin-resistant hippocampal neurons to generate enough neurotransmitter glutamate and (*b*) the effects of insulin as a neuromodulator itself in the hyperinsulinemic mice. Metabolic deficits in the hippocampal neurons could be a result of the observed impaired insulin signaling and the decreased translocation of GLUT3 and GLUT4 transporters, creating a deficiency in energy substrates required for neurotransmitter production. LTP elevates the post-synaptic density of AMPA receptors, whereas LTD leads to a decrease in its density. Insulin modulates glutamatergic neurotransmission by inducing GluR2 subunit phosphorylation in the AMPA receptor in the hippocampus, leading to endocytosis and thus decreases the post-synaptic excitatory ability^44^. Insulin signaling is also known to regulate learning and memory by promoting the translocation of GABA receptor to the plasma membrane^45^. In current work, lipoic acid treatment alone elevated I/O minimum value, and prevented HFD-induced I/O maximum value decrease. Lipoic acid treatment also prevented the decline in LTP in the HFD-feeding mice brain.

Overall, lipoic acid ameliorated HFD-induced obesity, insulin resistance, and brain glucose metabolism disruption in C56BL/6mice. Specifically, lipoic acid preserved insulin signaling by activating GLUT4 translocation to the plasma membrane, thus improving energy substrate availability for neuronal function, and consequently prevented HFD-induced reduction of synaptic plasticity. Therefore, lipoic acid could be considered a promising natural and nutritional intervention to prevent western diet-induced central nervous system insulin resistance and memory impairment.

## Materials and Methods

### Materials

R-sodium lipoic acid was a gift from GeroNova Research. A constant infusion of [1-^13^C]-glucose and [1,2-^13^C]-acetate was performed by using the ECONO gradient pump (Bio-Rad Laboratories, Hercules, CA, USA) and Rodent tail vein catheter and restraining apparatus (Braintree Scientific). Deuterium oxide (99.9%) and [1,2-^13^C]-acetate (99%) (Cambridge Isotope Laboratories, Andover, MA, USA), [1-^13^C]-glucose (99%) (Sigma-Aldrich, St Louis, MO, USA) were used for the NMR experiments. Chemicals of the purest grade were used for all assays. The primary antibodies against β-actin (SC-1616), GLUT3 (SC-74399), GLUT4 (sc-1608), Na^+^/K^+^-ATPase (SC-58628), and HRP-labeled secondary antibodies were obtained from Santa Cruz Biotechnology (Dallas, Texas, USA). Antibodies for Akt (9272) and p-Akt (Ser^473^) (9271) were purchased from Cell Signaling Technology (Danvers, MA, USA).

### Animals and Diet

C57BL/6J mice (Jackson Laboratories, Sacramento, CA, USA), aged for 3 months, and were housed under standard conditions in the animal facility (12/12 light-dark cycle, humidity at 50 ± 15%, temperature 22 ± 2 °C), and assigned to four groups based on either standard or high-fat diet diet (*n* = 10/group), and supplemention with or without lipoic acid (0.23% w/v) in drinking water for a total feeding period of 9 weeks. Both types of mice chow were purchased from Harlan-Teklad (now Envigo): standard diet (5053 Labdiet, 13% kcal from fat), and high-fat diet (60% kcal from fat; TD.06414). Body weight and food intake were recorded weekly to calculate caloric intake for each group. All procedures were approved by USC Department of Animal Resources and the Institutional Animal Care and Use Committee (protocol number: 11211). Anesthesia was used for all surgeries, and all efforts were made to minimize suffering. Five animals from each of the groups were used for PET imaging, NMR analysis, serum assays, and western blot analysis, while the remaining five from each group were used for LTP assays and western blot analysis post-*ex vivo* insulin stimulation.

### MicroPET/CT imaging

MicroPET imaging was performed as described before^10^ at the Molecular Imaging Center at the Department of Radiology, University of Southern California, with guidance from Dr. Li-Peng Yap. Blood-glucose levels were measured before the administration of the tracer to ensure that changes in glucose metabolism during [^18^F]- FDG-PET imaging were not due to differences in starting blood glucose levels, but represented intrinsic activity of the brain. After an overnight fasting period on drinking-water only, both lipoic acid-treated and control groups were sedated using 2% isoflurane by inhalation and administered the radiotracer 2-deoxy-2 [^18^F]-fluoro-D-glucose intravenously, followed by imaging in the Siemens MicroPET R4 scanner for 60 min. The mice, still sedated, were immediately transferred to the Siemens Inveon microCT scanner for 5 min for a CT scan. AMIDE (Free Software Foundation, Inc., Boston, MA, USA) was used to define region of interest and Standard uptake values (SUVs) was calculated based on the time, dose, and weight of the animal.

### Serum assays

Assays were performed as previously described^46^; fasting glucose levels were determined in mice that were fasted overnight. For glucose tolerance test, glucose levels were measured before intraperitoneal injection of glucose (1 g/kg body weight) and 15, 30, 60, and 120 min after the injection, using the OneTouch Ultra glucometer (Lifescan Benelux, Beerse, Belgium). Insulin levels were measured using the Elisa kit from Alpco, in serum obtained from orbital eye bleeding. Triglyceride levels were measured using a colorimetric plate test kit (Cayman Chemical, Michigan, USA).

### Western blot analyses

Whole brain tissue homogenates (n = 3 animals per group) were prepared using T-PER (Pierce Biotechnology, Rockford, IL, USA), while Cytosolic and membrane fractions were isolated using Mem-PER™ Plus Membrane Protein Extraction Kit (Pierce Biotechnology, Rockford, IL, USA) as per instructions provided by the manufacturer. Western blotting was performed as previous study described^47^. Extractions solubilized in SDS sample buffer, were separated by Laemmli SDS/PAGE, and transferred onto PVDF membranes. Appropriate primary antibodies (1:1000 dilution), and secondary antibodies (1:2000 dilution) were used to visualize immune-reactive bands using an enhanced chemiluminescence reagent.

### Long-Term Potentiation and I/O Curves and *Ex vivo* insulin stimulation

Electrophysiology experiments were performed as previously described^14^. Briefly, each animal was decapitated and coronal 350 µm thick hippocampal slices with surrounding cortical tissue was obtained. One slice was transferred to an interface-recording chamber and perfused with aCSF. Field excitatory postsynaptic potentials (fEPSPs) were recorded from stratum radiatum of CA1 using a glass pipette filled with 2 M NaCl in response to orthodromic stimulation of the Schaffer collateral-commissural projections. Pulses of 0.1 ms duration were delivered to the stimulating electrode every 20 s. Input/output (I/O) curves were generated using stimulus intensities ranging from 100–450 µA (in increments of 50 µA). Baseline fEPSP were evoked at 30–50% of maximal fEPSP in 20 s intervals. Long-term potentiation was induced at baseline intensity using Theta Burst Stimulation (TBS) consisting of ten trains of five 100 Hz stimulation repeated at 5 Hz. Field EPSP slope magnitude was calculated as the difference between two cursors, separated by 1 ms. Long-term potentiation values were expressed as a percentage of the average slope from the baseline recordings during the last 5 min of responses to TBS stimulator. *Ex vivo* insulin stimulating assays were performed as described in ref. 23: fresh brain slices then incubated with 10 nM insulin (Sigma, St. Louis, MO) for 10 min and washed with ice cold Artificial cerebrospinal fluid (aCSF). Tissues were lysed in lysis buffer and subjected to western blotting.

### ^13^C-Nuclear Magnetic Resonance and HPLC

Nuclear Magnetic Resonance (NMR) and high-performance liquid chromatography (HPLC) assays were performed as previously described^13^. Briefly, 0.6 M labeled solutions of glucose ([1-^13^C]-Glucose) and acetate ([1,2-^13^C]-Glucose) were prepared followed by restraining the mouse to be infused using a rotating tail injector (Braintree Scientific, Inc., MA, USA). The basal blood glucose levels were measured and a vein catheter (Braintree Scientific, Inc., MA, USA) was inserted. Initially, a bolus injection of the labeled glucose and acetate solution was injected followed by exponentially decreasing the amount of labeled glucose and acetate solution and finally, infusing at a constant rate for the different durations as specified (20, 60, and 150 minutes). At the end of the specified time, infusion was stopped, final blood glucose levels measured, and the mouse brain was immediately snap frozen using liquid nitrogen (minimizing the postmortem metabolic changes). A perchloric acid extraction was carried out and the final extract was used for NMR and HPLC analysis.

For NMR analysis, the final brain perchloric acid extract was thawed and mixed in appropriate proportion with D_2_O, 1.5 μL 1,4-dioxane (for reference peak), and a preservative (sodium azide). A Varian VNMRS 600 MHz instrument at 150.86 MHz was used for the ^13^C NMR analysis. A total of 7,312 scans were acquired at 25 °C. The details of the NMR acquisition, peak identification, and quantification of each peak along with the ^13^C-labelling patterns and interpretation have been detailed earlier^13^. For HPLC analysis, the perchloric acid extract was separated on a reverse-phase column with fluorometric detection after precolumn derivatization with o-phthalaldehyde and 2-mercaptoethanol. The percentage ^13^C enrichment of [4-^13^C]-Gln for example, was calculated from the concentration of [4-^13^C]-Gln (after correction for natural abundance ^13^C) and the total Glutamine (^12^C + ^13^C) concentration in each mouse.

### Metabolic Ratios

Relevant metabolic ratios have been calculated according to previous study^13^. Briefly, the metabolic ratios are calculated as following: (a) %Glycolytic activity = [3-^13^C]-Alanine/Mean value of ([3-^13^C]-Alanine in control group); TCA cycle activity = (2 × ([3-^13^C]-Glutamate- [1,2-^13^C]-Glutamate))/[4-^13^C]-Glutamate; (b) ^13^C-Glucose cycling ratio = ([3-^13^C]-Glutamate- [1,2-^13^C]-Glutamate)/[4-^13^C]-Glutamate or relative glutamine isotopomers; (c) ^13^C-acetate cycling ratio = [1,2-^13^C]-Glutamate/[4,5-^13^C]-Glutamate or relative glutamine isotopomers; (d) Transfer ratio from astrocytes to glutamatergic neurons = [4,5-^13^C]-Glutamate/[4,5-^13^C]- Glutamine; (e) Transfer ratio from astrocytes to GABAergic neurons = [4,5-^13^C]-Glutamate/[1, 2-^13^C]-GABA.

### Data analysis

Data are reported as means ± SD of at least three independent experiments. Significant differences between mean values were determined by Two-way ANOVA with and with diet (control/high-fat diet) and lipoic acid treatment (0 and 0.23% w/v in drinking water) as factors. Post-hoc analysis was performed using Student-Newman-Keuls correction for multiple comparison test. Means were considered to be statistically distinct if p < 0.05. All the F values and p values of ANOVA and multiple comparison of Table 1 and Figs 4 and 6 were shown in Supplemental Table 1.

## Electronic supplementary material

Supplementary Info file 1
