# Supplementary material for: Effects of Lipoic Acid on High-Fat Diet-Induced Alteration of Synaptic Plasticity and Brain Glucose Metabolism: A PET/CT and 13C-NMR Study
Source: Sci Rep. 2017 Jul 14;7:5391. doi: 10.1038/s41598-017-05217-z (PMC5511189; doi:10.1038/s41598-017-05217-z)
Supplement: Supplementary file 1 — Supplementary Info file 1 [file 41598_2017_5217_MOESM1_ESM.pdf]

EFFECTS OF LIPOIC ACID ON HIGH-FAT DIET-INDUCED ALTERATION OF SYNAPTIC PLASTICITY AND BRAIN GLUCOSE METABOLISM: A PET/CT AND ^{13}C-NMR STUDY

Zhigang Liu, Ishan Patil, Harsh Sancheti, Fei Yin, and Enrique Cadenas

Supplementary Information File 1

Supplementary Information File 1

Supplemental Figure 1

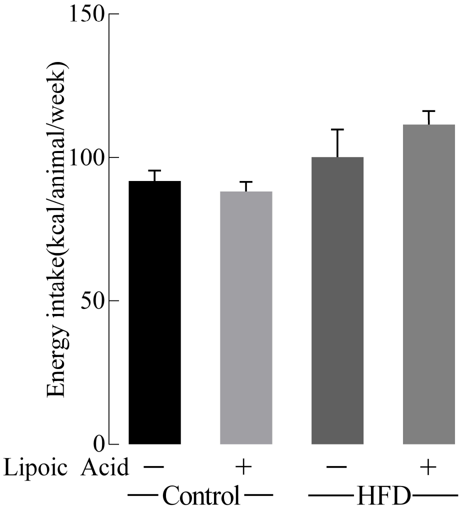

Supplemental Figure 1. Mice were fed with 9-week HFD or normal diet, with or without lipoic acid adimistration in drinking water (0.23% w/v) and the energy intake were monitored weekly and calculated at the end of the test.

Supplemental Figure 2

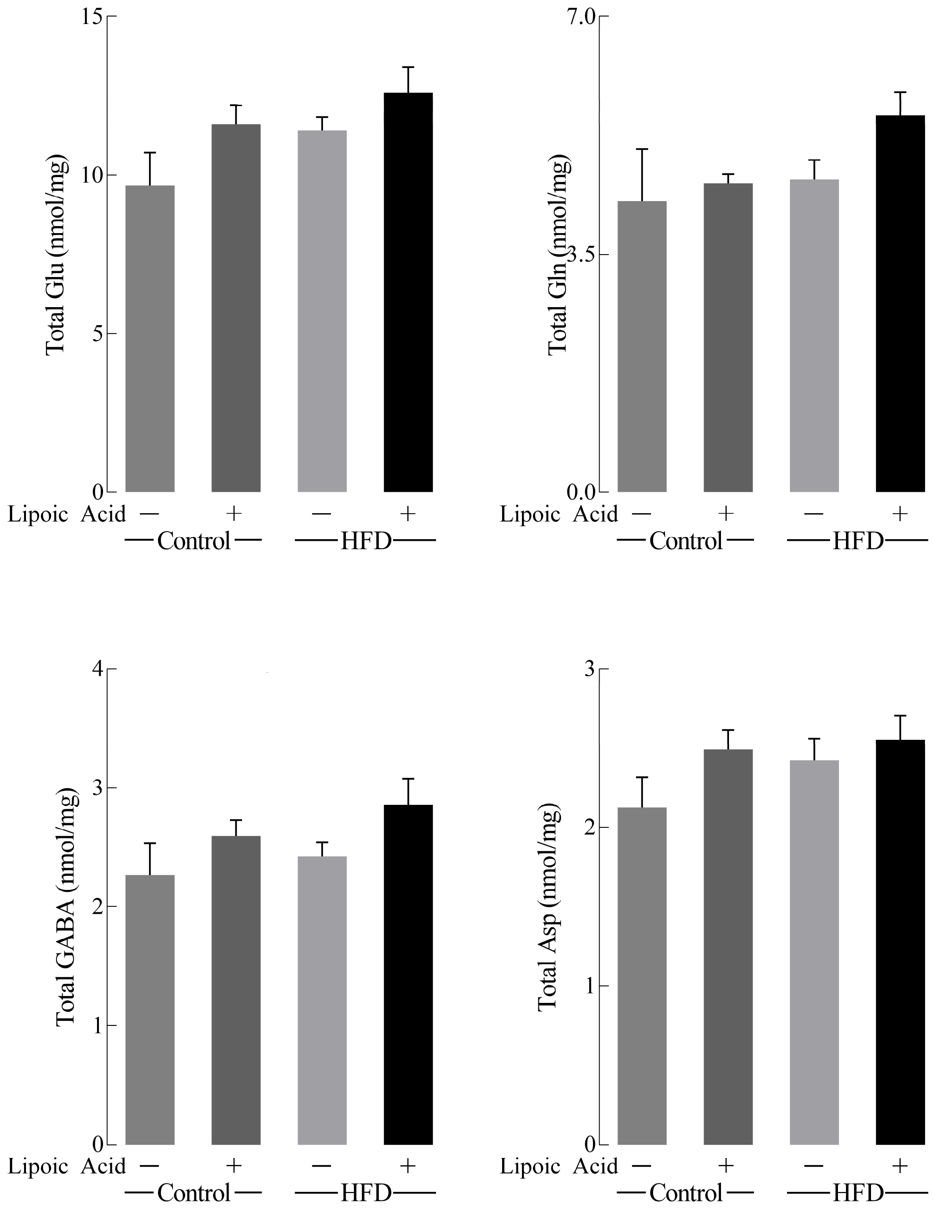

Supplemental Figure 2. Total metabolite levels (^{12}C+^{13}C) of Glu, Gln, GABA, and Asp in C57BL/6 mice ± lipoic acid feeding shown as mean nmol/mg brain tissue ± SEM.

Supplemental Figure 3

Original, full-length, and cropped images of western blots presented in Figure 3.

3A.

m-GLUT3

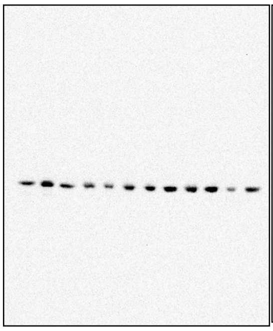

m-GLUT4

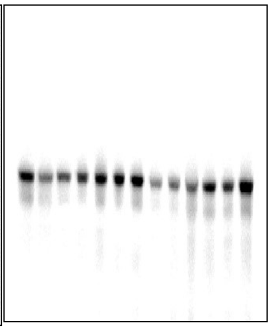

Na^+/K^+-ATPase

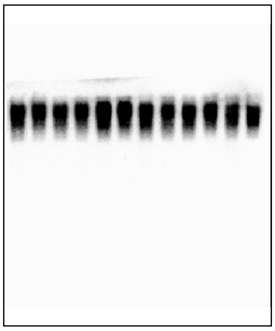

3B.

GLUT3

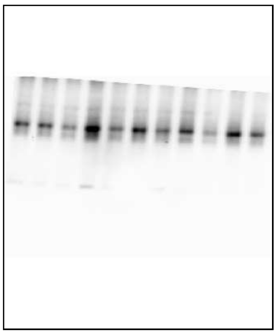

GLUT4

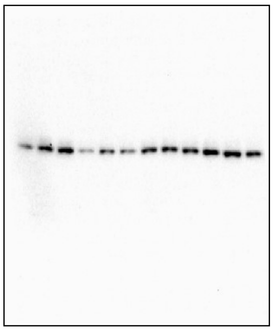

β-actin

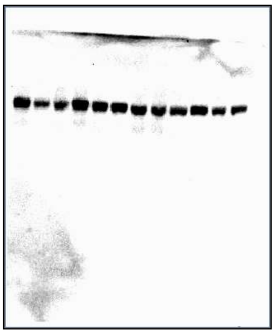

3C.

pAKT

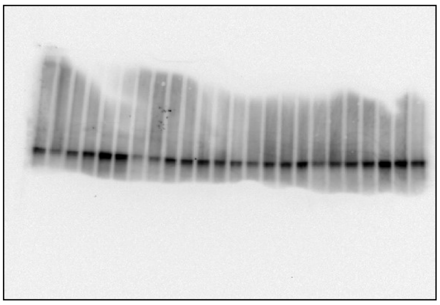

AKT

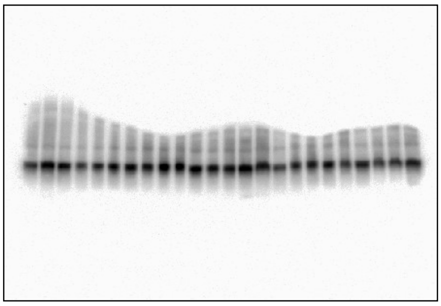

β-actin

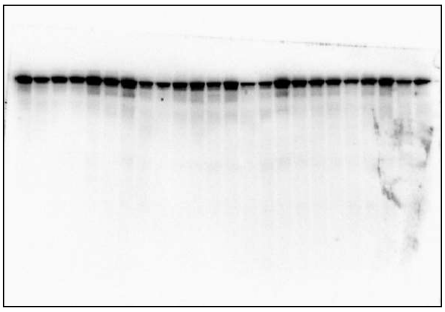

Supplemental Table 1

Table 1	TWO WAY ANOVA			Newman-Keuls multiple comparisons test	
[3-^{13}C]-Ala		F (DFn, DFd)	P value	**HFD vs. CON**	*
	Interaction	F (1, 15) = 4.972	P = 0.0415	LA vs. CON	ns
	HFD	F (1, 15) = 3.065	P = 0.1004	LA:HFD vs. CON	ns
	LA	F (1, 15) = 4.143	P = 0.0599	**LA vs. HFD**	*
				**LA:HFD vs. HFD**	*
				LA:HFD vs. LA	ns
[3-^{13}C]-Lac	Interaction	F (1, 15) = 0.1344	P = 0.7182	HFD vs CON	ns
	HFD	F (1, 15) = 2.065	P = 0.1679	LA vs CON	ns
	LA	F (1, 15) = 0.01922	P = 0.8913	LA:HFD vs. CON	ns
				LA vs HFD	ns
				LA:HFD vs HFD	ns
				LA:HFD vs. LA	ns
[4-^{13}C]-Glu	ANOVA table	F (DFn, DFd)	P value	HFD vs CON	ns
	Interaction	F (1, 15) = 0.1373	P = 0.7165	LA vs CON	ns
	HFD	F (1, 15) = 3.478	P = 0.0833	LA:HFD vs. CON	ns
	LA	F (1, 15) = 1.376	P = 0.2604	LA vs HFD	ns
				LA:HFD vs HFD	ns
				LA:HFD vs. LA	ns
[3-^{13}C]-Glu	Interaction	F (1, 15) = 0.03425	P = 0.8556	HFD vs CON	ns
	HFD	F (1, 15) = 2.804	P = 0.1147	LA vs CON	ns
	LA	F (1, 15) = 1.143	P = 0.3019	LA:HFD vs. CON	ns
				LA vs HFD	ns
				LA:HFD vs HFD	ns
				LA:HFD vs. LA	ns
[2-^{13}C]-Glu	Interaction	F (1, 15) = 0.1613	P = 0.6930	HFD vs CON	ns
	HFD	F (1, 15) = 3.254	P = 0.0890	LA vs CON	ns
	LA	F (1, 15) = 0.9936	P = 0.3328	LA:HFD vs. CON	ns
				LA vs HFD	ns
				LA:HFD vs HFD	ns
				LA:HFD vs. LA	ns
[4, 5-^{13}C]-Glu	Interaction	F (1, 15) = 0.1938	P = 0.6657	HFD vs CON	ns
	HFD	F (1, 15) = 5.679	P = 0.0299	LA vs CON	ns

	LA	F (1, 15) = 3.487	P = 0.0803	LA:HFD vs. CON	ns
				LA vs HFD	ns
				LA:HFD vs HFD	ns
				LA:HFD vs. LA	ns
[1, 2-^{13}C]-Glu	Interaction	F (1, 15) = 0.9158	P = 0.3528	HFD vs CON	ns
	HFD	F (1, 15) = 2.048	P = 0.1716	LA vs CON	ns
	LA	F (1, 15) = 5.878	P = 0.0275	LA:HFD vs. CON	ns
				LA vs HFD	ns
				LA:HFD vs HFD	ns
				LA:HFD vs. LA	ns
[2, 3-^{13}C]-Glu	Interaction	F (1, 15) = 0.01697	P = 0.8984	HFD vs CON	ns
	HFD	F (1, 15) = 0.2738	P = 0.6096	LA vs CON	ns
	LA	F (1, 15) = 0.006307	P = 0.9379	LA:HFD vs. CON	ns
				LA vs HFD	ns
				LA:HFD vs HFD	ns
				LA:HFD vs. LA	ns
[4-^{13}C]-Gln	Interaction	F (1, 15) = 0.0005792	P = 0.9811	HFD vs CON	ns
	HFD	F (1, 15) = 0.9444	P = 0.3466	LA vs CON	ns
	LA	F (1, 15) = 3.696	P = 0.0738	LA:HFD vs. CON	ns
				LA vs HFD	ns
				LA:HFD vs HFD	ns
				LA:HFD vs. LA	ns
[3-^{13}C]-Gln	Interaction	F (1, 15) = 0.3652	P = 0.5553	HFD vs CON	ns
	HFD	F (1, 15) = 2.471	P = 0.1383	LA vs CON	ns
	LA	F (1, 15) = 4.095	P = 0.0625	LA:HFD vs. CON	ns
				LA vs HFD	ns
				LA:HFD vs HFD	ns
				LA:HFD vs. LA	ns
[2-^{13}C]-Gln	Interaction	F (1, 15) = 0.4121	P = 0.5306	HFD vs CON	ns
	HFD	F (1, 15) = 0.7968	P = 0.3861	LA vs CON	ns
	LA	F (1, 15) = 0.6556	P = 0.4308	LA:HFD vs. CON	ns
				LA vs HFD	ns
				LA:HFD vs HFD	ns
				LA:HFD vs. LA	ns
[4, 5-^{13}C]-Gln	Interaction	F (1, 15) = 0.6086	P = 0.4474	**HFD vs CON**	*

	HFD	F (1, 15) = 3.397	P = 0.0852	LA vs CON	ns
	LA	F (1, 15) = 21.34	P = 0.0003	LA:HFD vs. CON	ns
				LA vs HFD	**
				LA:HFD vs HFD	ns
				LA:HFD vs. LA	**
[1, 2-^{13}C]-Gln	Interaction	F (1, 15) = 1.215	P = 0.2920	HFD vs CON	ns
	HFD	F (1, 15) = 3.347	P = 0.0923	LA vs CON	ns
	LA	F (1, 15) = 3.382	P = 0.0908	LA:HFD vs. CON	ns
				LA vs HFD	ns
				LA:HFD vs HFD	ns
				LA:HFD vs. LA	ns
[2, 3-^{13}C]-Gln#	Interaction	F (1, 15) = 1.749	P = 0.2072	**HFD vs CON**	**
	HFD	F (1, 15) = 1.594	P = 0.2274	LA vs CON	ns
	LA	F (1, 15) = 15.54	P = 0.0015	LA:HFD vs. CON	ns
				LA vs HFD	**
				LA:HFD vs HFD	ns
				LA:HFD vs. LA	ns
[4-^{13}C]-Asp	Interaction	F (1, 15) = 1.453	P = 0.2496	**HFD vs CON**	*
	HFD	F (1, 15) = 11.52	P = 0.0048	LA vs CON	ns
	LA	F (1, 15) = 4.053	P = 0.0653	LA:HFD vs. CON	ns
				LA vs HFD	**
				**LA:HFD vs HFD**	*
				LA:HFD vs. LA	ns
[3-^{13}C]-Asp	Interaction	F (1, 15) = 0.3693	P = 0.5519	HFD vs CON	ns
	HFD	F (1, 15) = 1.008	P = 0.3302	LA vs CON	ns
	LA	F (1, 15) = 0.008689	P = 0.9269	LA:HFD vs. CON	ns
				LA vs HFD	ns
				LA:HFD vs HFD	ns
				LA:HFD vs. LA	ns
[2-^{13}C]-Asp	Interaction	F (1, 15) = 0.6440	P = 0.4348	HFD vs CON	ns
	HFD	F (1, 15) = 1.012	P = 0.3304	LA vs CON	ns
	LA	F (1, 15) = 0.009164	P = 0.9250	LA:HFD vs. CON	ns
				LA vs HFD	ns
				LA:HFD vs HFD	ns
				LA:HFD vs. LA	ns
[1-^{13}C]-Asp	Interaction	F (1, 14) = 0.4090	P = 0.5328	HFD vs CON	ns

	HFD	F (1, 14) = 0.02480	P = 0.8771	LA vs CON	ns
	LA	F (1, 14) = 1.760	P = 0.2059	LA:HFD vs. CON	ns
				LA vs HFD	ns
				LA:HFD vs HFD	ns
				LA:HFD vs. LA	ns
[2, 3-^{13}C]-Asp	Interaction	F (1, 15) = 1.309	P = 0.2693	HFD vs CON	ns
	HFD	F (1, 15) = 4.115	P = 0.0595	LA vs CON	ns
	LA	F (1, 15) = 0.2951	P = 0.5944	LA:HFD vs. CON	ns
				LA vs HFD	ns
				LA:HFD vs HFD	ns
				LA:HFD vs. LA	ns
[3, 4-^{13}C]-Asp	Interaction	F (1, 15) = 0.4585	P = 0.5075	HFD vs CON	ns
	HFD	F (1, 15) = 1.153	P = 0.2979	LA vs CON	ns
	LA	F (1, 15) = 0.3561	P = 0.5585	LA:HFD vs. CON	ns
				LA vs HFD	ns
				LA:HFD vs HFD	ns
				LA:HFD vs. LA	ns
[4-^{13}C]-GABA	Interaction	F (1, 15) = 0.006026	P = 0.9391	HFD vs CON	ns
	HFD	F (1, 15) = 1.144	P = 0.3007	LA vs CON	ns
	LA	F (1, 15) = 0.1429	P = 0.7104	LA:HFD vs. CON	ns
				LA vs HFD	ns
				LA:HFD vs HFD	ns
				LA:HFD vs. LA	ns
[3-^{13}C]-GABA	Interaction	F (1, 15) = 5.412	P = 0.0368	**HFD vs CON**	**
	HFD	F (1, 15) = 5.761	P = 0.0321	LA vs CON	ns
	LA	F (1, 15) = 4.840	P = 0.0465	LA:HFD vs. CON	ns
				**LA vs HFD**	*
				**LA:HFD vs HFD**	**
				LA:HFD vs. LA	ns
[2-^{13}C]-GABA	Interaction	F (1, 15) = 6.714	P = 0.0205	**HFD vs CON**	*
	HFD	F (1, 15) = 2.524	P = 0.1330	LA vs CON	ns
	LA	F (1, 15) = 2.864	P = 0.1112	LA:HFD vs. CON	ns
				**LA vs HFD**	*
				**LA:HFD vs HFD**	*
				LA:HFD vs. LA	ns
[1-^{13}C]-GABA	Interaction	F (1, 14) = 1.632	P = 0.2222	HFD vs CON	ns

	HFD	F (1, 14) = 2.960	P = 0.1074	LA vs CON	ns
	LA	F (1, 14) = 0.01134	P = 0.9167	LA:HFD vs. CON	ns
				LA vs HFD	ns
				LA:HFD vs HFD	ns
				LA:HFD vs. LA	ns
[1,2-^{13}C]-GABA	Interaction	F (1, 16) = 0.6518	P = 0.4300	HFD vs CON	ns
	HFD	F (1, 16) = 4.670	P = 0.0444	LA vs CON	ns
	LA	F (1, 16) = 0.9282	P = 0.3481	LA:HFD vs. CON	ns
				LA vs HFD	ns
				LA:HFD vs HFD	ns
				LA:HFD vs. LA	ns
[2,3-^{13}C]-GABA	Interaction	F (1, 16) = 0.1831	P = 0.6738	HFD vs CON	ns
	HFD	F (1, 16) = 0.6463	P = 0.4319	LA vs CON	ns
	LA	F (1, 16) = 0.1725	P = 0.6828	LA:HFD vs. CON	ns
				LA vs HFD	ns
				LA:HFD vs HFD	ns
				LA:HFD vs. LA	ns
[3,4-^{13}C]-GABA	Interaction	F (1, 15) = 0.01264	P = 0.9122	HFD vs CON	ns
	HFD	F (1, 15) = 2.214	P = 0.1606	LA vs CON	ns
	LA	F (1, 15) = 1.762	P = 0.2072	LA:HFD vs. CON	ns
				LA vs HFD	ns
				LA:HFD vs HFD	ns
				LA:HFD vs. LA	ns
[4,6-^{13}C]-MI	Interaction	F (1, 15) = 2.092	P = 0.1652	HFD vs CON	ns
	HFD	F (1, 15) = 0.2718	P = 0.6085	LA vs CON	ns
	LA	F (1, 15) = 2.142	P = 0.1606	LA:HFD vs. CON	ns
				LA vs HFD	ns
				LA:HFD vs HFD	ns
				LA:HFD vs. LA	ns
[2-^{13}C]-MI	Interaction	F (1, 15) = 4.015	P = 0.0604	HFD vs CON	ns
	HFD	F (1, 15) = 0.1890	P = 0.6689	LA vs CON	ns
	LA	F (1, 15) = 0.5965	P = 0.4499	LA:HFD vs. CON	ns
				LA vs HFD	ns
				LA:HFD vs HFD	ns
				LA:HFD vs. LA	ns
[1,3-^{13}C]-MI	Interaction	F (1, 15) = 0.1929	P = 0.6657	HFD vs CON	ns

				LA:HFD vs. LA	ns
%[3-^{13}C]-Glu	ANOVA table	F (DFn, DFd)	P value	HFD vs.CON	ns
	Interaction	F (1, 15) = 1.489	P = 0.2425	LA vs.CON	ns
	HFD	F (1, 15) = 7.390	P = 0.0166	LA:HFD vs.CON	ns
	LA	F (1, 15) = 1.971	P = 0.1821	LA vs. HFD	ns
				LA:HFD vs. HFD	ns
				LA:HFD vs. LA	ns
%[2-^{13}C]-Glu	ANOVA table	F (DFn, DFd)	P value	HFD vs.CON	ns
	Interaction	F (1, 15) = 0.8934	P = 0.3606	LA vs.CON	ns
	HFD	F (1, 15) = 6.365	P = 0.0244	LA:HFD vs.CON	ns
	LA	F (1, 15) = 0.3222	P = 0.5793	LA vs. HFD	ns
				LA:HFD vs. HFD	ns
				LA:HFD vs. LA	ns
%[4, 5-^{13}C]-Glu	ANOVA table	F (DFn, DFd)	P value	HFD vs.CON	ns
	Interaction	F (1, 15) = 0.3575	P = 0.5602	LA vs.CON	ns
	HFD	F (1, 15) = 8.737	P = 0.0111	LA:HFD vs.CON	ns
	LA	F (1, 15) = 1.326	P = 0.2702	LA vs. HFD	ns
				LA:HFD vs. HFD	ns
				LA:HFD vs. LA	ns
%[1, 2-^{13}C]-Glu	ANOVA table	F (DFn, DFd)	P value	HFD vs.CON	ns
	Interaction	F (1, 16) = 0.9158	P = 0.3528	LA vs.CON	ns
	HFD	F (1, 16) = 2.048	P = 0.1716	LA:HFD vs.CON	ns
	LA	F (1, 16) = 5.878	P = 0.0275	LA vs. HFD	ns
				LA:HFD vs. HFD	ns
				LA:HFD vs. LA	ns
%[2, 3-^{13}C]-Glu#	ANOVA table	F (DFn, DFd)	P value	HFD vs.CON	ns
	Interaction	F (1, 15) = 0.2929	P = 0.5958	LA vs.CON	ns
	HFD	F (1, 15) = 4.667	P = 0.0463	LA:HFD vs.CON	ns
	LA	F (1, 15) = 1.016	P = 0.3284	LA vs. HFD	ns
				LA:HFD vs. HFD	ns
				LA:HFD vs. LA	ns
%[4-^{13}C]-Gln	ANOVA table	F (DFn, DFd)	P value	HFD vs.CON	ns
	Interaction	F (1, 15) = 0.5061	P = 0.4871	LA vs.CON	ns
	HFD	F (1, 15) = 5.051	P = 0.0391	LA:HFD vs.CON	ns
	LA	F (1, 15) = 0.3088	P = 0.5861	LA vs. HFD	ns
				LA:HFD vs. HFD	ns

	HFD	F (1, 15) = 0.001172	P = 0.9731	LA vs CON	ns
	LA	F (1, 15) = 0.2349	P = 0.6337	LA:HFD vs. CON	ns
				LA vs HFD	ns
				LA:HFD vs HFD	ns
				LA:HFD vs. LA	ns
[5-^{13}C]-MI	Interaction	F (1, 15) = 6.479	P = 0.0216	HFD vs CON	ns
	HFD	F (1, 15) = 0.4671	P = 0.5041	LA vs CON	ns
	LA	F (1, 15) = 2.119	P = 0.1648	LA:HFD vs. CON	ns
				LA vs HFD	ns
				LA:HFD vs HFD	ns
				LA:HFD vs. LA	ns
[6-^{13}C]-NAA	Interaction	F (1, 15) = 0.05496	P = 0.8176	HFD vs CON	ns
	HFD	F (1, 15) = 0.1240	P = 0.7294	LA vs CON	ns
	LA	F (1, 15) = 0.02992	P = 0.8648	LA:HFD vs. CON	ns
				LA vs HFD	ns
				LA:HFD vs HFD	ns
				LA:HFD vs. LA	ns
[3-^{13}C]-NAA	Interaction	F (1, 15) = 1.316	P = 0.2664	HFD vs CON	ns
	HFD	F (1, 15) = 3.751	P = 0.0686	LA vs CON	ns
	LA	F (1, 15) = 0.05959	P = 0.8099	LA:HFD vs. CON	ns
				LA vs HFD	ns
				LA:HFD vs HFD	ns
				LA:HFD vs. LA	ns
[2-^{13}C]-NAA	Interaction	F (1, 15) = 0.04492	P = 0.8347	HFD vs CON	ns
	HFD	F (1, 15) = 1.506	P = 0.2365	LA vs CON	ns
	LA	F (1, 15) = 1.020	P = 0.3266	LA:HFD vs. CON	ns
				LA vs HFD	ns
				LA:HFD vs HFD	ns
				LA:HFD vs. LA	ns
Figure 4	**TWO WAY ANOVA**			**Newman-Keuls multiple comparisons test**	
%[4-^{13}C]-Glu	ANOVA table	F (DFn, DFd)	P value	HFD vs.CON	ns
	Interaction	F (1, 15) = 1.515	P = 0.2402	LA vs.CON	ns
	HFD	F (1, 15) = 10.23	P = 0.0070	LA:HFD vs.CON	ns
	LA	F (1, 15) = 0.1621	P = 0.6938	LA vs. HFD	ns
				**LA:HFD vs. HFD**	*

				LA:HFD vs. LA	ns
%[3-^{13}C]-Gln	ANOVA table	F (DFn, DFd)	P value	HFD vs.CON	ns
	Interaction	F (1, 15) = 1.384	P = 0.2578	LA vs.CON	ns
	HFD	F (1, 15) = 6.106	P = 0.0259	LA:HFD vs.CON	ns
	LA	F (1, 15) = 0.1369	P = 0.7166	LA vs. HFD	ns
				LA:HFD vs. HFD	ns
				LA:HFD vs. LA	ns
%[2-^{13}C]-Gln	ANOVA table	F (DFn, DFd)	P value	HFD vs.CON	ns
	Interaction	F (1, 15) = 0.5232	P = 0.4799	LA vs.CON	ns
	HFD	F (1, 15) = 4.348	P = 0.0534	LA:HFD vs.CON	ns
	LA	F (1, 15) = 0.0006063	P = 0.9807	LA vs. HFD	ns
				LA:HFD vs. HFD	ns
				LA:HFD vs. LA	ns
%[4, 5-^{13}C]-Gln	ANOVA table	F (DFn, DFd)	P value	**HFD vs.CON**	*
	Interaction	F (1, 15) = 1.032	P = 0.3258	LA vs.CON	ns
	HFD	F (1, 15) = 6.684	P = 0.0207	LA:HFD vs.CON	ns
	LA	F (1, 15) = 13.12	P = 0.0025	LA vs. HFD	**
				**LA:HFD vs. HFD**	*
				LA:HFD vs. LA	ns
%[1, 2-^{13}C]-Gln	ANOVA table	F (DFn, DFd)	P value	**HFD vs.CON**	*
	Interaction	F (1, 15) = 4.654	P = 0.0503	LA vs.CON	ns
	HFD	F (1, 15) = 4.115	P = 0.0635	LA:HFD vs.CON	ns
	LA	F (1, 15) = 3.807	P = 0.0729	LA vs. HFD	*
				**LA:HFD vs. HFD**	*
				LA:HFD vs. LA	ns
%[2, 3-^{13}C]-Gln#	ANOVA table	F (DFn, DFd)	P value	**HFD vs.CON**	*
	Interaction	F (1, 15) = 1.300	P = 0.2733	LA vs.CON	ns
	HFD	F (1, 15) = 6.618	P = 0.0221	LA:HFD vs.CON	ns
	LA	F (1, 15) = 4.408	P = 0.0544	**LA vs. HFD**	*
				**LA:HFD vs. HFD**	*
				LA:HFD vs. LA	ns
%[4-^{13}C]-GABA	ANOVA table	F (DFn, DFd)	P value	HFD vs.CON	ns
	Interaction	F (1, 15) = 0.005719	P = 0.9406	LA vs.CON	ns
	HFD	F (1, 15) = 4.006	P = 0.0616	LA:HFD vs.CON	ns
	LA	F (1, 15) = 0.02576	P = 0.8744	LA vs. HFD	ns
				LA:HFD vs. HFD	ns

				LA:HFD vs. LA	ns
%[3-^{13}C]-GABA	ANOVA table	F (DFn, DFd)	P value	**HFD vs.CON**	**
	Interaction	F (1, 15) = 5.224	P = 0.0372	LA vs.CON	ns
	HFD	F (1, 15) = 10.31	P = 0.0058	LA:HFD vs.CON	ns
	LA	F (1, 15) = 4.362	P = 0.0542	**LA vs. HFD**	**
				**LA:HFD vs. HFD**	**
				LA:HFD vs. LA	ns
%[2-^{13}C]-GABA	ANOVA table	F (DFn, DFd)	P value	**HFD vs.CON**	**
	Interaction	F (1, 15) = 5.602	P = 0.0318	LA vs.CON	ns
	HFD	F (1, 15) = 8.948	P = 0.0091	LA:HFD vs.CON	ns
	LA	F (1, 15) = 4.730	P = 0.0460	**LA vs. HFD**	**
				**LA:HFD vs. HFD**	**
				LA:HFD vs. LA	ns
%[1-^{13}C]-GABA	ANOVA table	F (DFn, DFd)	P value	HFD vs.CON	ns
	Interaction	F (1, 14) = 1.765	P = 0.2109	LA vs.CON	ns
	HFD	F (1, 14) = 3.138	P = 0.1041	LA:HFD vs.CON	ns
	LA	F (1, 14) = 0.06131	P = 0.8090	LA vs. HFD	ns
				LA:HFD vs. HFD	ns
				LA:HFD vs. LA	ns
%[1,2-^{13}C]-GABA	ANOVA table	F (DFn, DFd)	P value	HFD vs.CON	ns
	Interaction	F (1, 15) = 0.1448	P = 0.7102	LA vs.CON	ns
	HFD	F (1, 15) = 4.725	P = 0.0505	LA:HFD vs.CON	ns
	LA	F (1, 15) = 1.055	P = 0.3246	LA vs. HFD	ns
				LA:HFD vs. HFD	ns
				LA:HFD vs. LA	ns
%[2,3-^{13}C]-GABA	ANOVA table	F (DFn, DFd)	P value	HFD vs.CON	ns
	Interaction	F (1, 15) = 0.2419	P = 0.6300	LA vs.CON	ns
	HFD	F (1, 15) = 0.4501	P = 0.5125	LA:HFD vs.CON	ns
	LA	F (1, 15) = 0.2254	P = 0.6418	LA vs. HFD	ns
				LA:HFD vs. HFD	ns
				LA:HFD vs. LA	ns
%[4-^{13}C]-Asp	ANOVA table	F (DFn, DFd)	P value	HFD vs. CON	ns
	Interaction	F (1, 15) = 0.08103	P = 0.7808	LA vs. CON	ns
	HFD	F (1, 15) = 5.253	P = 0.0408	LA:HFD vs. CON	ns
	LA	F (1, 15) = 3.157	P = 0.1010	LA vs. HFD	ns
				LA:HFD vs. HFD	ns

				LA:HFD vs.LA	ns
%[3-^{13}C]-Asp	ANOVA table	F (DFn, DFd)	P value	HFD vs.CON	ns
	Interaction	F (1, 15) = 0.04846	P = 0.9529	LA vs. CON	ns
	HFD	F (1, 15) = 2.872	P = 0.1140	LA:HFD vs. CON	ns
	LA	F (1, 15) = 1.294	P = 0.3073	LA vs. HFD	ns
				LA:HFD vs. HFD	ns
				LA:HFD vs.LA	ns
%[2-^{13}C]-Asp	ANOVA table	F (DFn, DFd)	P value	HFD vs. CON	ns
	Interaction	F (1, 15) = 0.01596	P = 0.9012	LA vs. CON	ns
	HFD	F (1, 15) = 1.679	P = 0.2147	LA:HFD vs. CON	ns
	LA	F (1, 15) = 0.1815	P = 0.6761	LA vs. HFD	ns
				LA:HFD vs. HFD	ns
				LA:HFD vs.LA	ns
%[1-^{13}C]-Asp	ANOVA table	F (DFn, DFd)	P value	HFD vs. CON	ns
	Interaction	F (1, 14) = 0.09941	P = 0.7569	LA vs. CON	ns
	HFD	F (1, 14) = 1.003	P = 0.3326	LA:HFD vs. CON	ns
	LA	F (1, 14) = 2.384	P = 0.1434	LA vs. HFD	ns
				LA:HFD vs. HFD	ns
				LA:HFD vs.LA	ns
%[1,2-^{13}C]-Asp	ANOVA table	F (DFn, DFd)	P value	HFD vs. CON	ns
	Interaction	F (1, 14) = 0.5522	P = 0.4697	LA vs. CON	ns
	HFD	F (1, 14) = 0.06892	P = 0.7967	LA:HFD vs. CON	ns
	LA	F (1, 14) = 0.04643	P = 0.8325	LA vs. HFD	ns
				LA:HFD vs. HFD	ns
				LA:HFD vs.LA	ns
%[2,3-^{13}C]-Asp	ANOVA table	F (DFn, DFd)	P value	HFD vs. CON	ns
	Interaction	F (1, 15) = 2.249	P = 0.1532	**LA vs. CON**	*
	HFD	F (1, 15) = 8.870	P = 0.0089	LA:HFD vs. CON	ns
	LA	F (1, 15) = 0.1691	P = 0.6864	LA vs. HFD	ns
				LA:HFD vs. HFD	ns
				LA:HFD vs.LA	ns
%[3,4-^{13}C]-Asp	ANOVA table	F (DFn, DFd)	P value	HFD vs. CON	ns
	Interaction	F (1, 15) = 1.064	P = 0.3159	LA vs. CON	ns
	HFD	F (1, 15) = 3.436	P = 0.0803	LA:HFD vs. CON	ns
	LA	F (1, 15) = 0.1951	P = 0.6640	LA vs. HFD	ns
				LA:HFD vs. HFD	ns

				LA:HFD vs.LA	ns
Figure 6	**TWO WAY ANOVA**			**Newman-Keuls multiple comparisons test**	
%Glycolity Acitivity		F (DFn, DFd)	P value	**HFD vs. CON**	*
	Interaction	F (1, 15) = 4.972	P = 0.0415	LA vs. CON	ns
	HFD	F (1, 15) = 3.065	P = 0.1004	LA:HFD vs. CON	ns
	LA	F (1, 15) = 4.143	P = 0.0599	LA vs. HFD	*
				**LA:HFD vs. HFD**	*
				LA:HFD vs. LA	ns
TCA Cycle Activity		F (DFn, DFd)	P value	**HFD vs. CON**	*
	Interaction	F (1, 15) = 6.921	P = 0.0208	LA vs. CON	ns
	HFD	F (1, 15) = 0.2142	P = 0.6512	LA:HFD vs. CON	ns
	LA	F (1, 15) = 4.424	P = 0.0555	LA vs. HFD	ns
				LA:HFD vs. HFD	ns
				LA:HFD vs. LA	ns
^{13}C Glucose Cycling Glutamine	Interaction	F (1, 15) = 0.03147	P = 0.8619	HFD vs. CON	ns
	HFD	F (1, 15) = 0.05630	P = 0.8161	LA vs. CON	ns
	LA	F (1, 15) = 0.03393	P = 0.8567	LA:HFD vs. CON	ns
				LA vs. HFD	ns
				LA:HFD vs. HFD	ns
				LA:HFD vs. LA	ns
^{13}C Glucose Cycling Glutamate	Interaction	F (1, 15) = 6.921	P = 0.0208	**HFD vs. CON**	*
	HFD	F (1, 15) = 0.2142	P = 0.6512	LA vs. CON	ns
	LA	F (1, 15) = 4.424	P = 0.0555	LA:HFD vs. CON	ns
				LA vs. HFD	ns
				LA:HFD vs. HFD	ns
				LA:HFD vs. LA	ns
^{13}C Acetate Cycling Ratio Glutamate	Interaction	F (1, 15) = 2.902	P = 0.1091	HFD vs. CON	ns
	HFD	F (1, 15) = 1.801	P = 0.1996	LA vs. CON	ns
	LA	F (1, 15) = 0.2003	P = 0.6609	LA:HFD vs. CON	ns
				LA vs. HFD	ns
				LA:HFD vs. HFD	ns
				LA:HFD vs. LA	ns
^{13}C Acetate Cycling Ratio Glutamine	Interaction	F (1, 14) = 0.08376	P = 0.7762	HFD vs. CON	ns
	HFD	F (1, 14) = 0.01694	P = 0.8982	LA vs. CON	ns
	LA	F (1, 14) = 0.08765	P = 0.7712	LA:HFD vs. CON	ns

				LA vs. HFD	ns
				LA:HFD vs. HFD	ns
				LA:HFD vs. LA	ns
Astrocytes-Glu-Neurons Ratios	Interaction	F (1, 15) = 1.167	P = 0.3055	**HFD vs. CON**	*
	HFD	F (1, 15) = 1.129	P = 0.3131	LA vs. CON	ns
	LA	F (1, 15) = 15.86	P = 0.0026	**LA:HFD vs. CON**	**
				LA vs. HFD	ns
				LA:HFD vs. HFD	ns
				LA:HFD vs. LA	ns
Astrocytes-GABA-Neurons Ratios Astrocytes-Glu-Neurons Ratios	Interaction	F (1, 15) = 3.734	P = 0.0773	**HFD vs. CON**	*
	HFD	F (1, 15) = 0.05036	P = 0.8262	LA vs. CON	ns
	LA	F (1, 15) = 9.830	P = 0.0086	LA:HFD vs. CON	ns
				LA vs. HFD	ns
				LA:HFD vs. HFD	ns
				LA:HFD vs. LA	ns

These data were analyzed by by Two-way ANOVA with diet (control/high-fat diet) and lipoic acid treatment (0 and 0.23% w/v in drinking water) as factors. Post-hoc analysis was performed using Student-Newman-Keuls correction for multiple comparison test. CON stands for control group, LA stands for lipoic acid treatment group, HFD stands for high-fat diet-feeding group, LA:HFD stands for high-fat diet with lipoic acid treatment group, ns stands for no significance, $*p \leq 0.05$ $**p \leq 0.01$, and [2,3-^{13}C]-Gln/Glu$^{\#}$ stand for [2,3 and 3,4-^{13}C]-Gln/Glu, respectively.
